# Epitope-specific airway-resident CD4^+^ T cell dynamics during experimental human RSV infection

**DOI:** 10.1172/JCI131696

**Published:** 2019-12-09

**Authors:** Aleks Guvenel, Agnieszka Jozwik, Stephanie Ascough, Seng Kuong Ung, Suzanna Paterson, Mohini Kalyan, Zoe Gardener, Emma Bergstrom, Satwik Kar, Maximillian S. Habibi, Allan Paras, Jie Zhu, Mirae Park, Jaideep Dhariwal, Mark Almond, Ernie H.C. Wong, Annemarie Sykes, Jerico Del Rosario, Maria-Belen Trujillo-Torralbo, Patrick Mallia, John Sidney, Bjoern Peters, Onn Min Kon, Alessandro Sette, Sebastian L. Johnston, Peter J. Openshaw, Christopher Chiu

**Affiliations:** 1 National Heart and Lung Institute and; 2 Department of Infectious Disease, Imperial College London, London, United Kingdom.; 3 Centre for Infectious Disease, Division of Vaccine Discovery, La Jolla Institute for Allergy and Immunology, La Jolla, California, USA.; 4 Department of Medicine, UCSD, La Jolla, California, USA.

**Keywords:** Immunology, Infectious disease, Adaptive immunity, T cells

## Abstract

**BACKGROUND:**

Respiratory syncytial virus (RSV) is an important cause of acute pulmonary disease and one of the last remaining major infections of childhood for which there is no vaccine. CD4^+^ T cells play a key role in antiviral immunity, but they have been little studied in the human lung.

**METHODS:**

Healthy adult volunteers were inoculated i.n. with RSV A Memphis 37. CD4^+^ T cells in blood and the lower airway were analyzed by flow cytometry and immunohistochemistry. Bronchial soluble mediators were measured using quantitative PCR and MesoScale Discovery. Epitope mapping was performed by IFN-γ ELISpot screening, confirmed by in vitro MHC binding.

**RESULTS:**

Activated CD4^+^ T cell frequencies in bronchoalveolar lavage correlated strongly with local C-X-C motif chemokine 10 levels. Thirty-nine epitopes were identified, predominantly toward the 3′ end of the viral genome. Five novel MHC II tetramers were made using an immunodominant EFYQSTCSAVSKGYL (F-EFY) epitope restricted to HLA-DR4, -DR9, and -DR11 (combined allelic frequency: 15% in Europeans) and G-DDF restricted to HLA-DPA1*01:03/DPB1*02:01 and -DPA1*01:03/DPB1*04:01 (allelic frequency: 55%). Tetramer labeling revealed enrichment of resident memory CD4^+^ T (Trm) cells in the lower airway; these Trm cells displayed progressive differentiation, downregulation of costimulatory molecules, and elevated CXCR3 expression as infection evolved.

**CONCLUSIONS:**

Human infection challenge provides a unique opportunity to study the breadth of specificity and dynamics of RSV-specific T-cell responses in the target organ, allowing the precise investigation of Trm recognizing novel viral antigens over time. The new tools that we describe enable precise tracking of RSV-specific CD4^+^ cells, potentially accelerating the development of effective vaccines.

**TRIAL REGISTRATION:**

ClinicalTrials.gov NCT02755948.

**FUNDING:**

Medical Research Council, Wellcome Trust, National Institute for Health Research.

## Graphical abstract

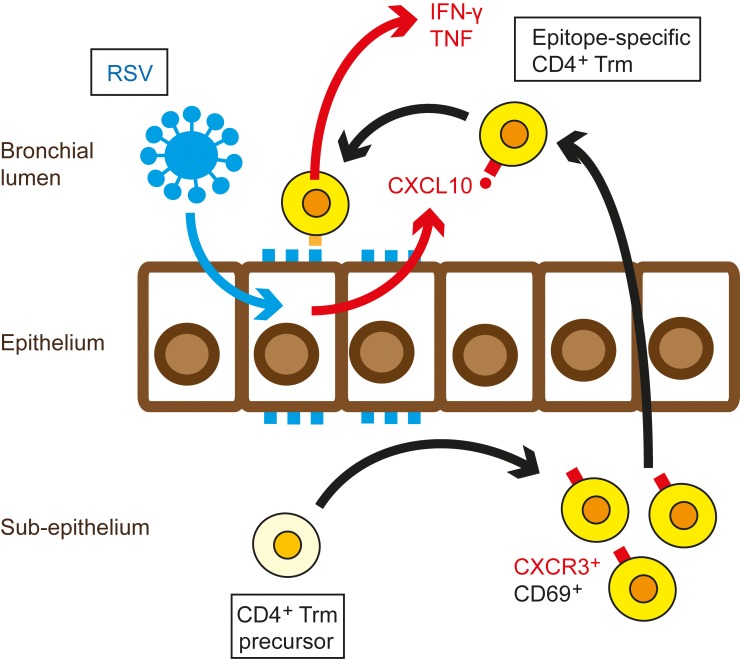

## Introduction

T cells are a critical component of long-term adaptive immunity and have been shown to be essential for clearance of intracellular pathogens (1, 2), the development of high-affinity antibodies (3), and generation of long-lived adaptive immune responses (4, 5). Nevertheless, the role of T cells for most acute infections in humans remains poorly understood. This finding is particularly true for the diverse range of CD4^+^ T helper cells. Major hurdles that hinder the investigation of CD4^+^ T cells include their low frequencies in peripheral blood, the diversity of HLA haplotypes and thus variability of responses between subjects (6), and the promiscuity of MHC class II epitopes as well as their generally low binding affinities (7–9). Therefore, for many acute infections, class II–restricted epitopes are poorly characterized, and few detailed investigations of the temporal dynamics and location of antigen-specific CD4^+^ T cell responses have been reported.

Among the major causes of acute infection is respiratory syncytial virus (RSV), a pathogen of global importance affecting millions around the world each year. Primary infection with RSV is the most prevalent cause of infant hospitalization around the world and leads to an estimated 3.2 million hospital admissions and 59,000 in-hospital deaths with acute lower respiratory infection in children younger than 5 years of age (10). In adults, RSV causes recurrent symptomatic reinfection throughout life despite relatively little antigenic change in circulating strains, probably due to virus-induced immunomodulation and immune evasion (11). In older individuals, this results in severe disease and mortality particularly in those with comorbidities (12, 13). Despite decades of research, treatment remains supportive and the only available prophylaxis (the mAb palivizumab) is out of reach of the majority of those who are at risk of severe illness (14). Recent advances in our understanding of newly described targets for humoral immunity in the RSV F protein have radically transformed vaccine development in this area (15), but major bottlenecks still exist that limit the generation of effective, long-term protection in the various high-risk groups, with several late-stage clinical trials of RSV vaccine candidates having recently failed (16, 17).

CD4^+^ T cells in animal models of RSV have been shown to be important in viral clearance, with CD4^+^ T cell depletion leading to prolonged viral shedding (18, 19). In adoptive transfer models, they have also been implicated in increased disease severity as well as being involved in the dysregulated immunity that may have been responsible for vaccine-enhanced disease with formalin-inactivated RSV (20, 21). In other model systems, T follicular helper cells have also been shown to be essential for immunoglobulin class switching (e.g., to mucosal IgA), selection of high-affinity clones undergoing somatic hypermutation, and help for B cell differentiation into long-lived plasma cells and memory B cells (22). CD4^+^ T cells are therefore likely to be essential to long-lived vaccine-induced response.

Because respiratory viral infections are usually confined to the respiratory tract, circulating T cells may not represent the most relevant protective population (23). Recently, a subset of noncirculating memory T cells has been described that is specialized to protect sites of pathogen entry (24). These resident memory T (Trm) cells are not only poised for rapid killing on virus re-encounter but also exhibit innate-like sensing and inflammatory functions (25). During murine influenza, lung Trm cells induced by respiratory challenge confer greater protection than spleen-derived cells of the same specificity while also mediating heterosubtypic immunity (26, 27). As well as direct antiviral activity, CD4^+^ T cell help has been shown to be required for the effective commitment of long-lived memory CD8^+^ T cells including CD8^+^ Trm cells in the lung (28).

Although our knowledge of RSV-specific CD8^+^ T cells in both the periphery and the respiratory tract is gradually improving, understanding of human CD4^+^ T cells in RSV infection remains minimal, despite their likely importance (29, 30). In view of their central role in coordinating adaptive immunity, further knowledge of T helper cell function will be key to understanding why only partial immunity is induced by RSV infection and critical for effective vaccines (31). To address this knowledge gap, we analyzed the kinetics, specificities, and functionality of the cell-mediated response to RSV in experimentally infected human volunteers. At peak T cell expansion, we characterized epitope-specific responses to the entire RSV proteome, identifying immunodominant MHC class II–restricted epitopes and generating tetramers that provided sensitive tools with which to probe rare RSV-specific CD4^+^ T cells in both blood and lung. Using the particular strengths of human challenge infection, we have thus shown the breadth of CD4^+^ T cell targets in human RSV and demonstrate correlates of CD4^+^ T cell recruitment to the lung.

## Results

### CD4^+^ T cells infiltrate the lower respiratory mucosa following RSV challenge and peak 10 days after infection.

**Figure 1:**
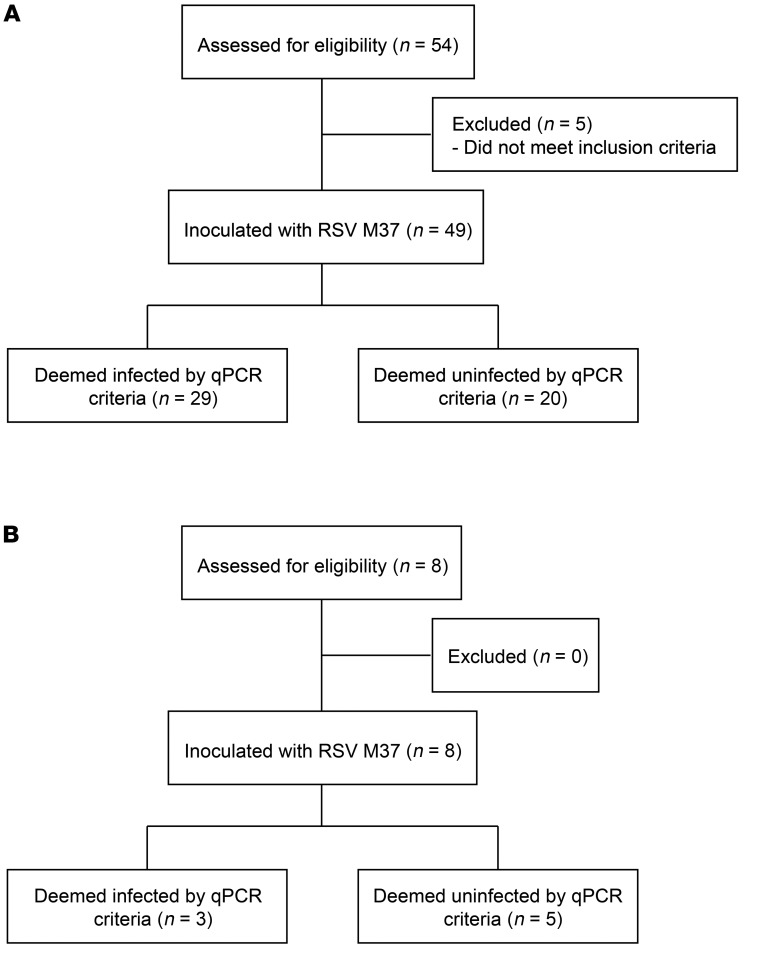
Flow diagram outlining study design and participating subjects. (**A**) Healthy adult volunteers (*n* = 49) were enrolled and inoculated with RSV M37 for polyclonal CD4^+^ T cell analysis and epitope discovery. (**B**) A second cohort (*n* = 8) was enrolled for tetramer analysis of RSV-specific CD4^+^ T cells.

To investigate the cell-mediated response to RSV infection, 49 healthy adults were challenged with RSV Memphis 37 (M37) i.n. as previously described (32). Of these, 29 (51%) became infected by quantitative PCR (qPCR) criteria (see Methods) (Figure 1A). An additional 8 individuals (of whom 3/8 became infected) were challenged separately to validate findings from the first cohort (Figure 1B). Demographic characteristics are shown in Supplemental Table 1 (supplemental material available online with this article: https://doi.org/10.1172/JCI131696DS1). Consistent with previous studies, viral load became detectable in infected individuals after an approximately 3-day incubation period, rising to peak at around days 7 to 8 after inoculation (mean, 2.76 log10 copies/mL ± SEM 0.322; Supplemental Figure 1A). This was associated with primarily upper respiratory tract symptoms (Supplemental Figure 1, B and C). Symptoms significantly correlated with cumulative nasal viral load (AUC *r*_s_ = 0.6129, *P* = 0.0009; Supplemental Figure 1D). Our previous studies showed that preinfection nasal IgA levels correlate with protection from infection with RSV, but that systemic serum-neutralizing antibodies are clearly less protective in an experimental challenge (32). These data were supported by similar (although nonsignificant) findings in this smaller cohort (Supplemental Figure 2).

**Figure 2:**
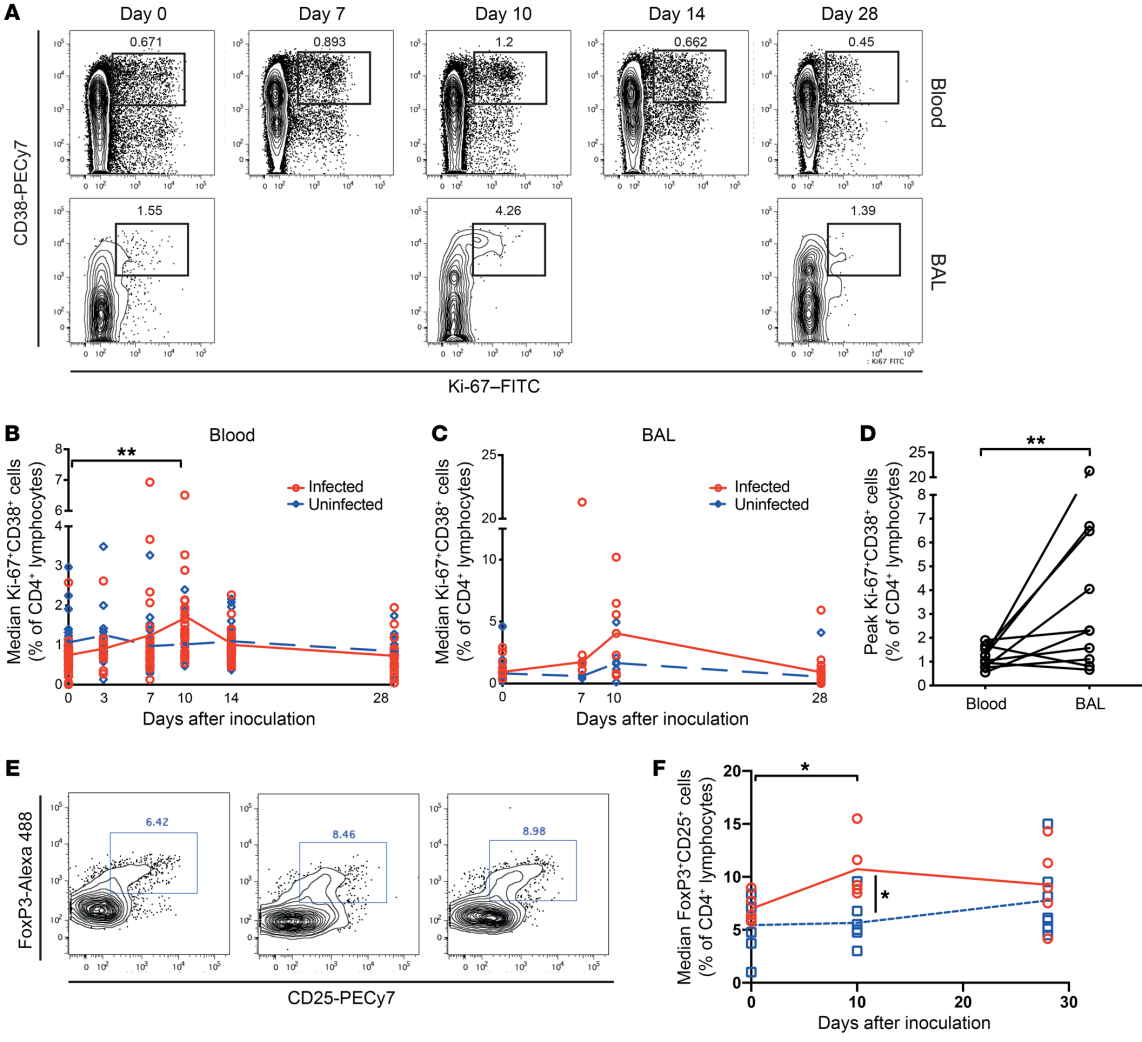
Enrichment of activated and regulatory CD4^+^ T cells in the lower airway during RSV infection. (**A**) Whole blood (*n* = 49) and BAL (*n* = 24) samples were stained with anti-CD3, -CD4, -CD8, -CD38, and –Ki-67 for analysis by flow cytometry. Plots are gated on CD3^+^CD4^+^ lymphocytes. One representative infected subject is shown for blood (upper panels) and BAL (lower panels). Median and individual data points of Ki-67^+^CD38^+^CD4^+^ T cells in the (**B**) blood and (**C**) BAL of infected (PCR^+^, red) or uninfected (PCR^–^, blue) volunteers are shown. Tests of the 5 a priori hypotheses were conducted by Wilcoxon’s signed-rank test with Bonferroni-adjusted α levels of 0.01 (**P < 0.001). (**D**) Frequencies of Ki-67^+^CD38^+^ cells on day 10 after infection are compared between paired blood and BAL samples in infected individuals (*n* = 12). Tests of the 5 a priori hypotheses were conducted by Wilcoxon’s signed-rank test with Bonferroni-adjusted α levels of 0.01 with no statistically significant differences seen. (**E**) Whole blood and BAL samples were stained with anti-CD3, -CD4, -FoxP3, and -CD25. One representative infected BAL sample is shown gated on CD3^+^CD4^+^ lymphocytes. (**F**) Mean and individual data points of FoxP3^+^CD25^+^CD4^+^ T cells in the blood and BAL of infected (PCR^+^, red circles) or uninfected (PCR^–^, blue squares) volunteers are shown. *P* values for Wilcoxon’s signed-rank (intragroup) and Mann-Whitney tests (intergroup) are shown. *P < 0.05.

To track T cell activation and proliferation, whole blood samples were stained with anti–Ki-67 and CD38 for flow cytometric analysis of CD4^+^ T cells before inoculation (day 0) and 3, 7, 10, 14, and 28 days after the challenge (Figure 2A). In blood, the frequency of activated CD4^+^ T cells increased between 7 and 10 days after infection, coinciding with viral clearance. Ki-67^+^CD38^+^CD4^+^ T cells peaked around day 10 (median, 1.33%; IQR, 1.87–1.08), after which they returned to baseline frequencies on disease resolution (median, 0.67%; IQR, 0.757–0.449; Figure 2B). Although the magnitude of the proliferative response was modest, activated and proliferating CD4^+^ T cells were significantly more frequent than in those challenged individuals who remained “uninfected.”

A subset of participants (*n* = 24) underwent bronchoscopy with bronchoalveolar lavage (BAL) to sample the lower airway on days 0, 7 to 10, and 28 after inoculation; 12 of these individuals (50%) became infected following viral inoculation. Proliferation and activation of CD4^+^ T cells in BAL was similar to that in blood, although there was substantial variability between individuals (Figure 2, A and C). Within individuals, activated CD4^+^ T cells following infection were significantly more frequent in BAL than blood (mean, 4.73% ± SEM 1.97 vs. 1.17% ± SEM 0.137; *P* = 0.0273; Figure 2D). In 1 volunteer, more than 20% of CD4^+^ T cells in the BAL displayed an activated phenotype. Analysis of CD4^+^ T cells with a regulatory phenotype (FoxP3^+^CD25^+^ Tregs) showed additional divergence between blood and BAL (Figure 2, E and F). Similarly to total CD4^+^ T cells, the frequency of Tregs was significantly greater in BAL throughout. However, although no change in Treg frequencies in blood were seen over the course of infection (Supplemental Figure 3), in BAL those same individuals showed significantly increased Treg frequencies on day 10 after infection compared with preinfection frequencies (*P* = 0.0317) and compared with the uninfected group at the same time point (*P* = 0.0177) (Figure 2F).

**Figure 3:**
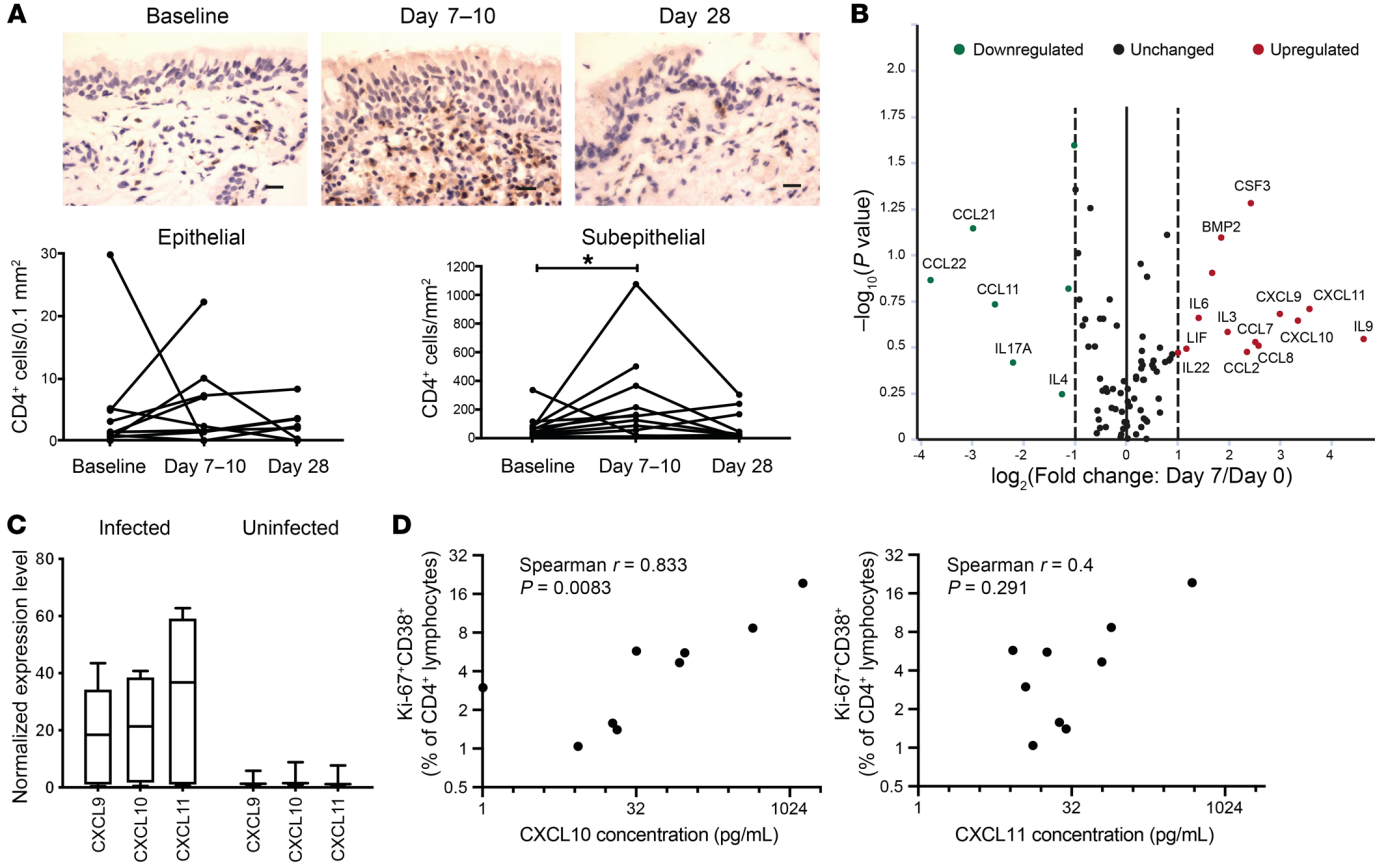
Activated CD4^+^ T cell frequencies in the lower airway correlate strongly with CXCL10 expression. (**A**) CD4^+^ cells (brown) were identified in bronchial biopsies (*n* = 12) by immunohistochemistry and enumerated in infected individuals. Scale bars: 20 μm. Individual data points are presented as number of positive cells per square millimeter of subepithelium or per 0.1 mm^2^ of epithelium. *P < 0.05 by Wilcoxon’s signed-rank test. (**B**) Differential cytokine and chemokine gene expression in bronchial brushings 7 days after infection compared with preinfection was analyzed by RT2 Profiler qPCR array. Greater than 2-fold upregulated (red) and downregulated (green) genes are shown. (**C**) Mean ± SEM gene expression levels of CXCL9, CXCL10, and CXCL11 are shown in infected (red) and uninfected (green) individuals. (**D**) Spearman’s correlations between bronchial mucosal lining fluid CXCL10 or CXCL11 concentrations and the frequency of Ki-67^+^CD38^+^CD4^+^ T cells in BAL are shown.

Immunohistochemical staining of bronchial biopsies also showed significant infiltration of CD4^+^ cells particularly in the subepithelium at the acute time points (Figure 3A). Again, wide variation was demonstrable in the number of CD4^+^ cells recruited to the airway mucosa. To investigate potential determinants of this wide variability in CD4^+^ T cell frequencies in the airway, markers of antigen load and disease severity were analyzed. Although viral load has been reported to be a driver of disease severity (33), the frequencies of activated CD4^+^ T cells in blood (Supplemental Figure 4A) and BAL (Supplemental Figure 4B), as well as the number of CD4^+^ cells in bronchial biopsies (Supplemental Figure 4C) correlated poorly with viral load as measured in nasal lavage. Furthermore, there was no correlation between the number of subepithelial CD4^+^ cells and the frequency of activated CD4^+^ T cells in BAL (Supplemental Figure 4D), suggesting that recruitment of CD4^+^ T cells to the airway may involve a more complex mechanism than antigen abundance.

Proinflammatory cytokines and chemokines are known to regulate inflammation and recruitment of immune cells. Bronchial brushings obtained before and 7 days after infection were therefore analyzed using a qPCR array of soluble mediator genes to screen for associations with CD4^+^ T cell recruitment (Figure 3B and Supplemental Table 2). Compared with the preinfection time point, 14 genes were upregulated on day 7 with a greater than 2-fold change. Due to the limited sample size, none was statistically significant and thus no multiple testing correction was applied to the illustrative data. Among the most upregulated were IL-9 (produced by CD4^+^ T cells and mast cells, with pleiotropic effects on a variety of cell types); CCL2, CCL7, and CCL8, which promote inflammatory recruitment of monocytes/macrophages among others; and the known T cell chemoattractants C-X-C motif chemokine 9 (CXCL9), CXCL10, and CXCL11. None of these latter 3 chemokines was upregulated in volunteers who had been challenged with virus but resisted infection (Figure 3C). To test their correlations with recruitment of activated CD4^+^ T cells to the airway, CXCL10 and CXC11 levels in bronchial lining fluid were analyzed by Meso Scale Discovery (Figure 3D). Although CXCL11 levels only showed a trend toward association with Ki-67^+^CD38^+^CD4^+^ T cells in BAL, CXCL10 correlated strongly with the frequency of activated BAL CD4^+^ T cells at the acute time point. Thus, human CD4^+^ T cells are likely recruited to the airway during RSV infection as part of a coordinated inflammatory mediator response involving CXCL10.

### CD4^+^ T cells in BAL display a highly differentiated tissue-tropic phenotype.

**Figure 4:**
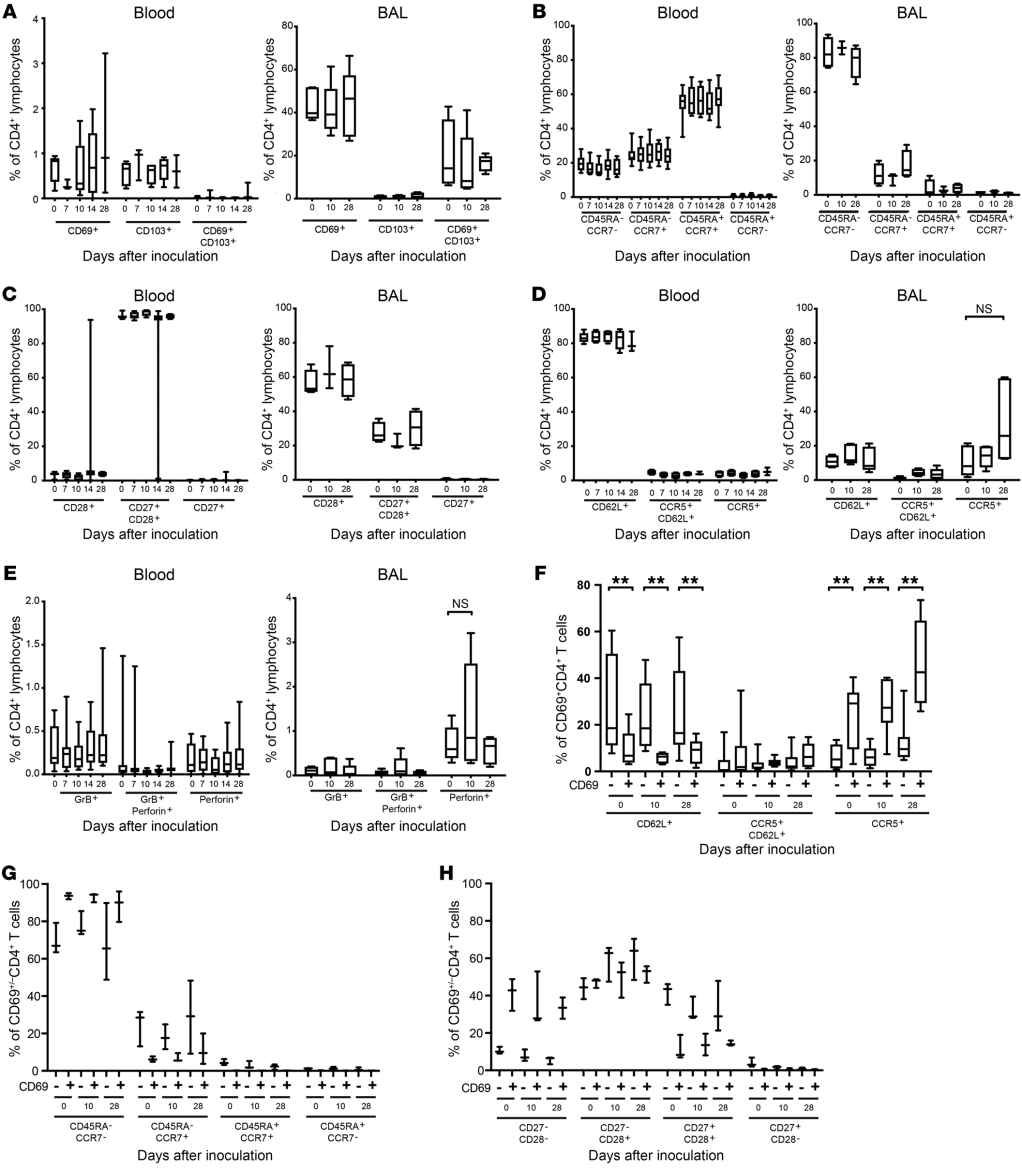
CD69^+^ resident memory CD4^+^ T cells in BAL exhibit advanced differentiation. Whole blood/PBMCs (*n* = 10) and BAL (*n* = 5) from individuals infected with RSV were costained with anti-CD3, -CD4, and phenotypic markers and then analyzed by flow cytometry. (**A**) CD69 and CD103 as canonical markers of resident memory T cells are shown in blood and BAL from infected volunteers. Mean ± SEM frequencies are shown. (**B**) Memory markers CD45RA and CCR7, (**C**) costimulatory markers CD27 and CD28, (**D**) homing markers CCR5 and CD62L, and (**E**) cytotoxicity markers perforin and granzyme B are shown in blood and BAL. In CD69^+^ (Trm) and CD69^–^ (non-Trm) subsets from BAL, frequencies of (**F**) CCR5 and CD62L, (**G**) CD45RA and CCR7, and (**H**) CD27 and CD28-expressing CD4^+^ T cell are shown. *P* values for paired *t* test are shown. **P < 0.01.

The only consistently recognized CD4^+^ Trm cell marker is CD69, and the phenotypic characteristics of CD4^+^ Trm cells over the course of infection are unclear. In peripheral blood, we found that less than 1% of CD4^+^ T cells expressed CD69 and almost none expressed both CD69 and CD103 (Figure 4A and Supplemental Figure 5A). Trm cells coexpressing CD69 and CD103 dominated the CD8^+^ T cell pool in BAL (34), but only a minority (<20%) of CD4^+^ T cells in the airway expressed both Trm markers, and approximately half of the remaining cells expressed CD69 alone (Figure 4A). None of these populations altered significantly in frequency during the course of infection.

T cell differentiation leads to phenotypic changes that are associated with antiviral function. To analyze the phenotype of these CD4^+^ T cell populations, whole blood and BAL samples from PCR^+^ subjects were analyzed for markers of costimulation (CD27 and CD28), memory subsets (CD45RA and CCR7), homing (CD62L and CCR5), and cytotoxicity (perforin and granzyme B; Figure 4, B–E, and Supplemental Figure 5, B–E). CD4^+^ T cells exhibited distinct phenotypic differences between blood and BAL: cells in blood predominantly displayed a naive (CD45RA^+^CCR7^+^; mean 54.41% ± SEM 2.71 on day 0) phenotype with the remaining cells divided between effector (CD45RA^–^CCR7^–^) and central memory T cells (CD45RA^–^CCR7^+^; mean 82.9% ± SEM 4.46), whereas those in BAL preinfection expressed an effector memory phenotype with almost no naive cells and fewer central memory T cells (mean, 11.8% ± SEM 3.09) (Figure 4B and Supplemental Figure 5B). Consistent with the predominance of naive cells, more than 95% of CD4^+^ T cells in blood coexpressed CD27 and CD28 at every time point. In BAL, however, approximately 60% of CD4^+^ T cells had downregulated CD27, indicative of more advanced differentiation (Figure 4C and Supplemental Figure 5C). Although the majority of CD4^+^ cells in blood expressed the lymphoid homing marker CD62L, in BAL, this was expressed in only approximately 10% of CD4^+^ T cells (Figure 4D and Supplemental Figure 5D). In contrast, low frequencies of CCR5-expressing CD4^+^ T cells (mean, 3.93% ± SEM 0.667) were found in blood with higher frequencies expressing CCR5 in BAL (mean, 10.8% ± SEM 3.93). Previous studies in human influenza challenge show a correlation between cytotoxic CD4^+^ T cells in peripheral blood preinfection and reduced disease severity (2). However, perforin- and granzyme B–expressing CD4^+^ T cells were found at low frequencies in both blood and BAL at all time points (Figure 4E and Supplemental Figure 5E). Although there was a trend toward higher frequencies of perforin^+^CD4^+^ T cells in BAL and a trend toward a decrease in CD4^+^ T cells expressing cytotoxicity markers in peripheral blood during the acute time points, none of these was statistically significant.

Because CD4^+^ T cells in BAL were a mixture of CD69^+^ and CD69^–^ cells (containing Trm and non-Trm cells, respectively), we further analyzed the heterogeneity of these populations and their expression of differentiation markers. At all time points, the proportion of CD69^+^CD4^+^ T cells expressing CCR5 was significantly higher than among CD69^–^CD4^+^ T cells (Figure 4F). Conversely, CD69^–^CD4^+^ T cells expressing CD62L were significantly more frequent than in the CD69^+^ subset, implying that Trm cells are preferentially attracted to sites of inflammation, whereas non-Trm cells are more likely to have the capacity to circulate through lymphoid tissues. Furthermore, CD69^+^CD4^+^ T cells were more likely to display an effector memory T cell phenotype (Figure 4G), with higher proportions of these cells having downregulated both CD27 and CD28 (Figure 4H), indicating a more advanced differentiation status.

Analysis of these phenotypic subsets revealed marked differences in predominant populations between blood and BAL, but the modest RSV-specific response in the context of a largely resting total CD4^+^ population failed to be reflected in any statistically significant changes over the course of infection. However, phenotypic divergence suggestive of more advanced differentiation in BAL CD4^+^ T cells was shown.

### RSV-specific CD4^+^ T cells in adults exclusively express type 1 cytokines.

**Figure 5:**
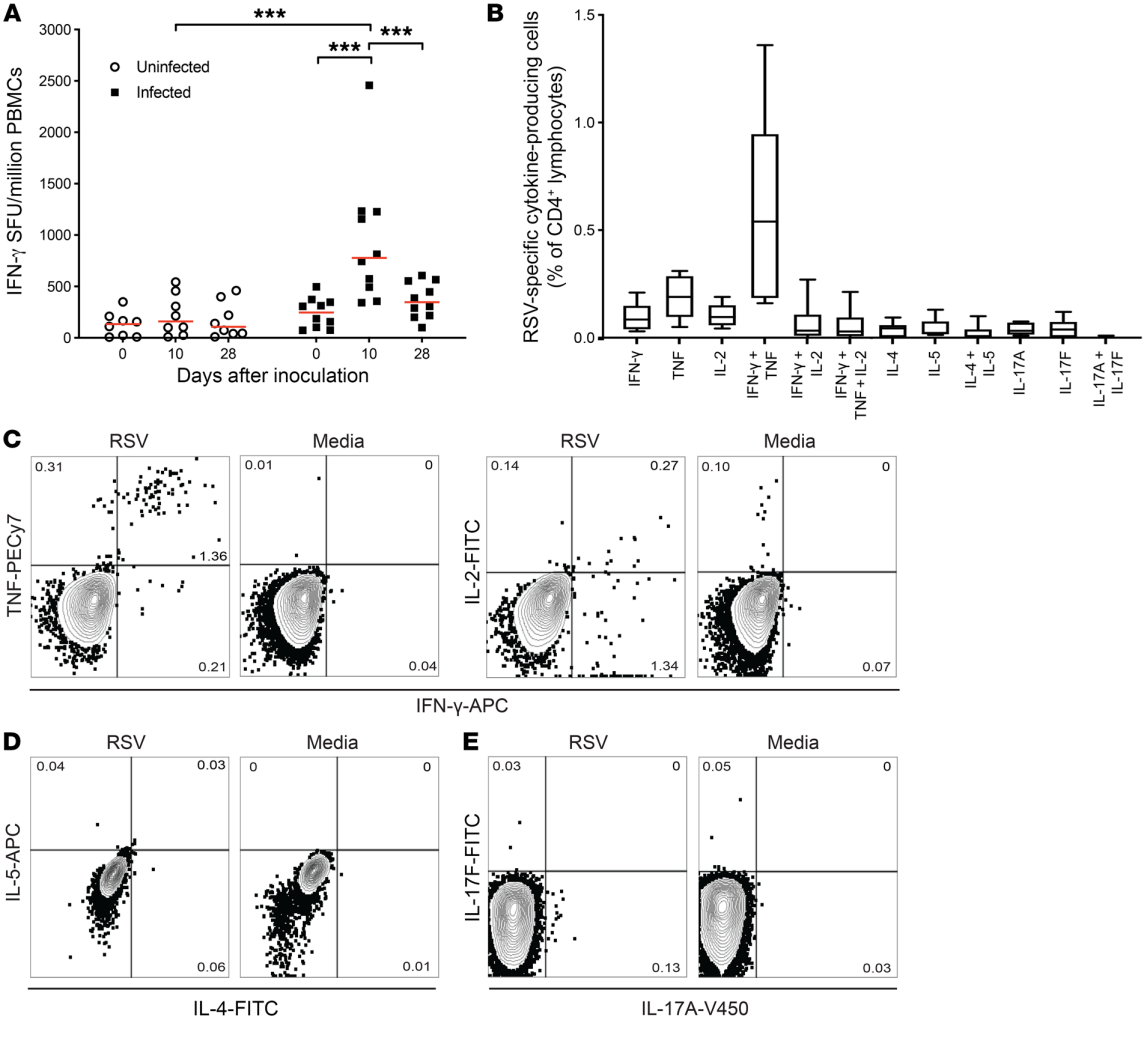
RSV-specific CD4^+^ T cells primarily express 1 or 2 Th1 cytokines. (**A**) PBMCs from RSV-challenged subjects (*n* = 18) were assayed by IFN-γ ELISpot stimulated with whole RSV. Median and individual data points are shown with *P* values for 2-tailed Mann-Whitney test comparing infected and uninfected groups, and 2-tailed Wilcoxon’s matched-pairs tests comparing time points. ***P < 0.001. (**B**) Median and IQR of T helper cell subsets expressing different cytokines are shown. (**C**–**E**) PBMCs from infected subjects (*n* = 10) on day 10 after infection were cultured in vitro with live RSV or media for 24 hours and Brefeldin A added 4 hours before staining with fixable viability dye and with antibodies against CD3, CD4, and (**C**) IFN-γ, IL-2, and TNF; (**D**) IL-4, IL-5; or (**E**) IL-17A and IL-17F. Representative FACS plots from a single participant are shown.

To determine the magnitude of the virus-specific response to RSV, cytokine production by antigen-specific T cells was induced with whole virus at an optimized MOI (Supplemental Figure 6A). By ELISpot, IFN-γ–producing RSV-specific T cells from infected subjects were shown to be significantly more frequent on day 10 compared with baseline (*P* < 0.001) and convalescence (*P* < 0.001; Figure 5A), consistent with the kinetics of total CD4^+^ T cell activation. By depletion of CD8^+^ T cells from PBMCs, CD4^+^ T cells were shown to contribute 41.2% (median; IQR, 41.7–30.6) of this RSV-specific IFN-γ response (Supplemental Figure 6B).

Dysregulated T cell responses are believed to contribute to RSV-induced immunopathology (35). To test whether Th2 or Th17 cytokines are expressed by T helper cells in the adult immune response to RSV, PBMCs isolated on day 10 from infected participants were stimulated with whole virus and analyzed by flow cytometry (Figure 5, B–E). In contrast to some reports of RSV bronchiolitis in infants and formalin-inactivated RSV vaccine–enhanced disease, CD4^+^ T cells responding to RSV almost exclusively expressed type 1 cytokines (Figure 5, B and C). The majority of Th1 cells coexpressed IFN-γ and TNF (mean, 0.6% ± SEM 0.182), which contrasted with the total CD4^+^ T cell population where the majority of cells produced IFN-γ alone on PMA/ionomycin stimulation (Supplemental Figure 6C). There was little evidence of multiple (≥3) cytokine production characteristic of the polyfunctional T cells elsewhere associated with the most potent antiviral efficacy, and there was neither increased expression of IL-4 and IL-5 compared with preinfection or unstimulated controls (Th2; Figure 5D) nor IL-17A and IL-17F (Th17; Figure 5E).

In studies of influenza challenge, CD4^+^ memory T cells in blood were associated with a reduction in symptoms on subsequent infection (2). However, there was no difference in the frequency of preexisting IFN-γ–producing RSV-specific memory T cells between individuals who subsequently became infected or remained uninfected following challenge (Supplemental Figure 7A), no correlation between preexisting RSV-specific memory T cells and disease severity as measured by viral load (Supplemental Figure 7B), and no association between viral load and magnitude of RSV-specific T cell response in peripheral blood (Supplemental Figure 7C). Thus, RSV-specific CD4^+^ T cells were induced following infection and peaked around day 10, expressing type 1 cytokines with limited polyfunctionality, but no correlation with protection was detected.

### Immunodominant CD4^+^ T cell epitopes are found in F and G proteins.

To define the breadth of antigen-specific responses and optimal T cell targets for vaccine-induced immunity, freshly isolated PBMCs from day 0 before infection, and days 10 and 28 after infection were stimulated using peptide pools spanning the RSV proteome and detected by IFN-γ ELISpot.

**Figure 6:**
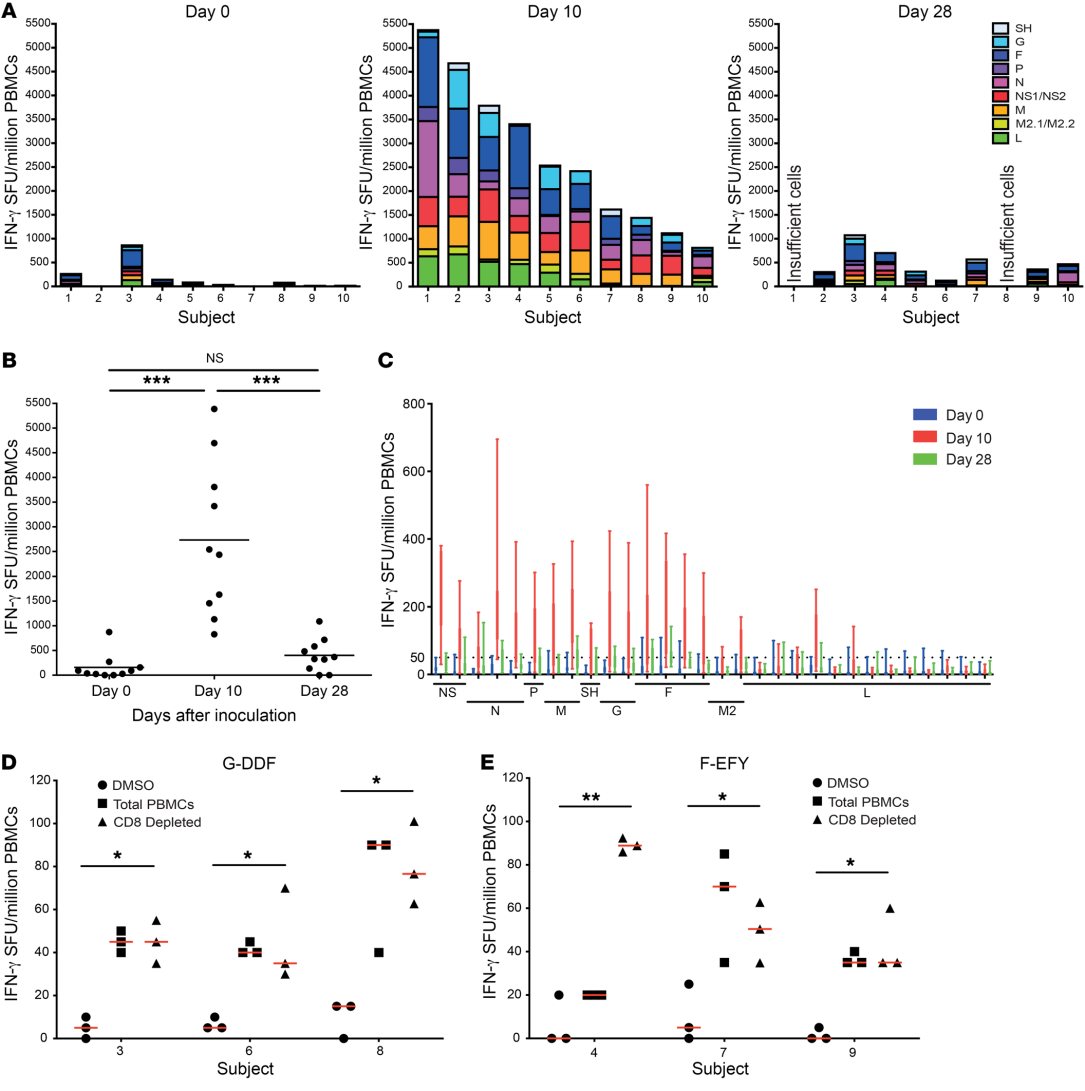
Immunodominant CD4^+^ T cell epitopes are found in the surface F and G proteins. Fresh PBMCs from individuals infected with RSV (*n* = 10) were assayed by IFN-γ ELISpot using overlapping peptides covering the RSV proteome. (**A**) ELISpot responses to peptide pools on days 0, 10, and 28 after infection are arranged according to the originating protein. (**B**) Total ELISpot responses to peptide pools on days 0, 10, and 28 after infection are shown. Median and individual counts are shown with *P* values for 2-tailed Wilcoxon’s matched-pairs tests comparing time points. (**C**) Median and IQR ELISpot responses to each peptide pool are shown in RSV genome order. (**D**) Median and IQR ELISpot responses to G-DDF (*n* = 3) and (**E**) F-EFY (*n* = 3) peptides are shown. *P* values are for 2-tailed Wilcoxon’s matched-pairs tests. *P < 0.05, **P < 0.01, ***P < 0.001.

The resultant estimates of antigen-specific T cell frequencies against each RSV protein revealed substantial interindividual variability in the size and breadth of the T cell populations both at rest and during infection (Figure 6, A and B). Prior to infection, fewer than 100 spots per million PBMCs were seen in most (7 out of 10) individuals, highlighting their relative scarcity at rest (median 64 spots/million; IQR, 186–17) (Figure 6A). However, by day 10 after infection, these had expanded up to 50-fold to range from 829 spots/million PBMCs to 5388 spots/million PBMCs (median, 2488 spots/million; IQR, 4029–1376), depending on the individual. Again, there was no correlation between memory T cell frequencies and reduced disease severity or size of peak T cell response and viral load (Supplemental Figure 7, D and E). Following infection, virus-specific T cells fell rapidly leaving an enlarged memory pool on day 28 (median, 423 spots/million; IQR, 684–320).

Analysis at the level of individual proteins revealed the majority of IFN-γ–producing T cell responses were directed against pools corresponding to the internal proteins NS1/2, N, P, and M and the surface proteins F and G (Figure 6C), that is, proteins derived from the 3′ end of the genome, with few responses against the L protein (despite its large size).

Peptide pools that induced T cell responses were next deconvoluted to identify candidate epitopes. Individual peptides that stimulated a positive response (>50 IFN-γ spots/million PBMCs) in at least 2 subjects were subsequently investigated and their conservation with the prototypic laboratory RSV A A2 strain tested (Table 1). Twelve of the 39 peptides (30.8%) thus identified had not been previously described as RSV epitope candidates. Of those that had been reported, the ones that mapped to internal proteins all had been shown to be recognized by CD8^+^ T cells (34, 36). Following infection, responses to the internal protein epitopes M-LGA, M-LLV, and N-VML induced the highest IFN-γ^+^ T cell responses. This finding was consistent with our previous work showing them as immunodominant CD8^+^ T cell epitopes (34).

In contrast, epitope candidates within the surface proteins have been primarily described as inducing CD4^+^ T cell responses (Table 1). Compared with epitope-specific CD8^+^ T cell responses, where the median response against the most immunodominant class I–restricted epitopes was 231 spots/million (IQR, 577–138), the size of response against any individual peptide in F (median 33 spots/million; IQR, 40–20) or G (median, 34 spots/million; IQR, 45–27) was significantly smaller. Nevertheless, 2 epitopes (DDFHFEVFNFVPCSI [G-DDF] and EFYQSTCSAVSKGYL [F-EFY]) were identified as inducing the highest frequency responses in at least 3 subjects (Figure 6, D and E). Depletion of CD8^+^ T cells from day-10 PBMCs had no effect on the frequency of IFN-γ–producing cell frequency, indicating that these responses were derived from CD4^+^ T cells.

### F-EFY and G-DDF stimulate CD4^+^ T cell proliferation and cytokine production on a wide HLA background.

To determine the HLA class II allelic restriction of the F-EFY and G-DDF epitopes, the Immune Epitope Database MHC-II binding prediction tool was used to screen for predicted binding affinities. F-EFY was predicted to bind multiple HLA-DRB1 molecules with high affinity (consensus percentile < 20), with the top binders being *04:01, *09:01, and *11:01 (Supplemental Table 3). In contrast, G-DDF was predicted to bind DP molecules DPA1*01:03/DPB1*02:01 and DPA1*01/DPB1*04:01. These results aligned with the HLA haplotypes of the responding subjects (Supplemental Table 4) and were confirmed by competitive MHC binding assays that showed F-EFY binding to DRB1*04:01 (12 nM), DRB1*09:01 (460 nM), and DRB1*11:01 (681 nM) with high affinity (Table 2). Similarly, G-DDF was also shown to bind DPA1*01:03/DPB1*02:01 and DPA1*01/DPB1*04:01 at high affinity with measured affinities of 13 nM and 1.8 nM, respectively.

**Figure 7:**
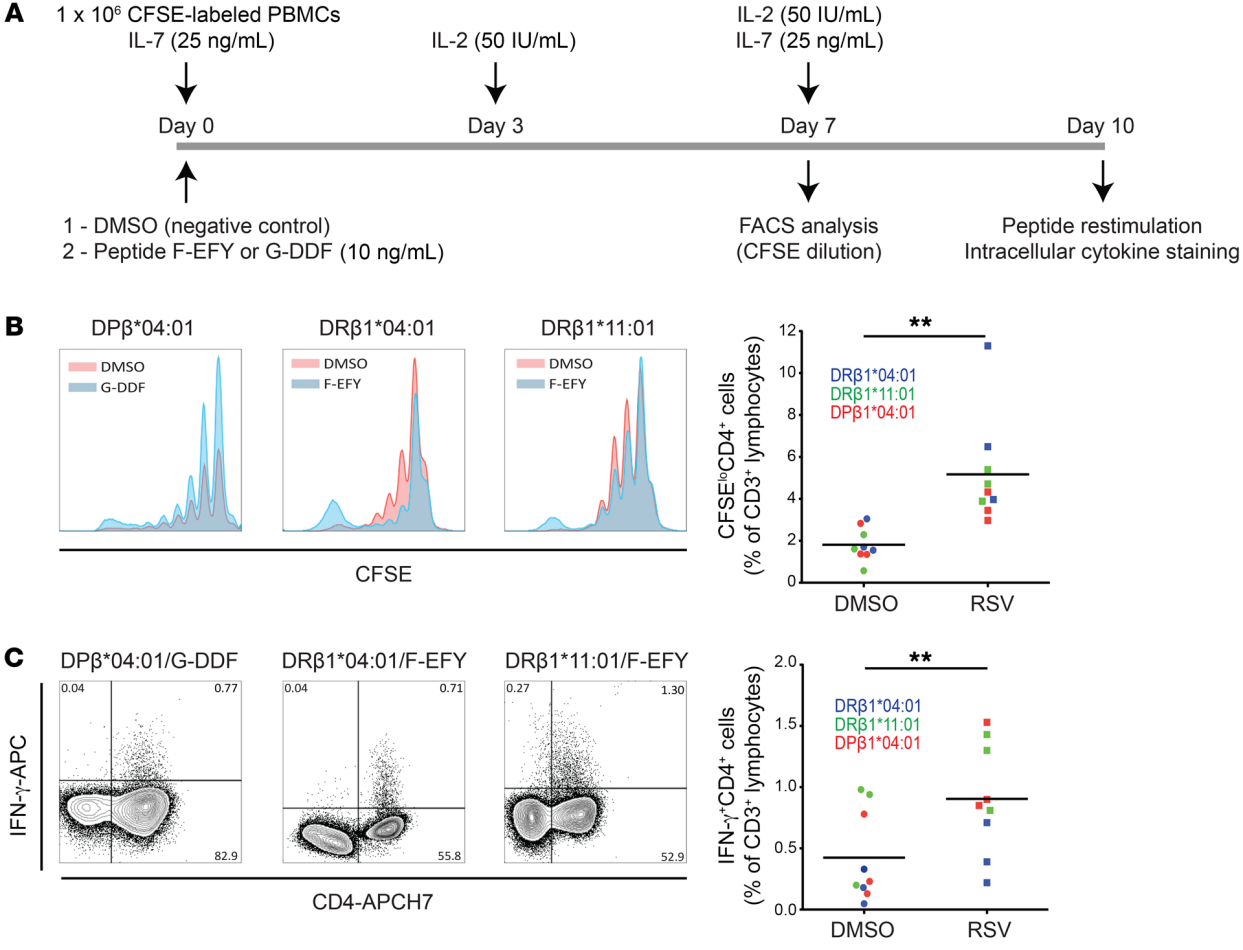
Immunodominant epitopes from F and G proteins induce proliferation and cytokine production in vitro. (**A**) CFSE-stained PBMCs from uninfected HLA-matched donors were cultured with F-EFY, G-DDF, or DMSO and supplemented with IL-2 and IL-7. (**B**) CFSE dilution was analyzed by flow cytometry (*n* = 9). PBMCs from 6 representative donors expressing differing HLA alleles are shown. Blue histograms show cultures with peptide epitopes and red show DMSO negative controls. Median and individual data points of frequencies of CFSE^lo^ CD4^+^ T cells after stimulation are plotted. (**C**) PBMCs (*n* = 9) were restimulated after 10 days with peptide epitopes and assayed by intracellular staining for IFN-γ and flow cytometry. Medians and individual data points of IFN-γ^+^CD4^+^ T cell frequencies are plotted. Two-tailed Mann-Whitney test was used to compare RSV- and DMSO-only responses (**P < 0.01).

Having confirmed HLA binding of the epitopes, further validation of their capacity to induce CD4^+^ T cell responses was conducted in vitro. Preinfection PBMC samples from matched subjects with the most common HLAs (DRB1*04:01, DRB1*11:01, and DPB1*04:01) were labeled with CFSE and cultured with F-EFY or G-DDF plus IL-2 and IL-7 supplementation for 10 days (Figure 7A). Resulting proliferation revealed enhanced CFSE dilution in the presence of the HLA-matched peptide (Figure 7B). The addition of peptide epitope thus specifically enhanced maximal (>6 divisions) proliferation with significantly higher CFSE-negative CD4^+^ T cell frequencies compared with negative (DMSO) control. Additionally, following 10-day culture, PBMCs were restimulated with peptide epitope and stained for intracellular cytokines (Figure 7C). Culture with the epitopes led to expansion of CD4^+^ T cells capable of producing IFN-γ on restimulation, resulting in a significantly higher frequency of epitope-specific CD4^+^ T cells compared with those cultured with DMSO alone. F-EFY and G-DDF were therefore characterized as CD4^+^ T cell epitopes presented by DRB1 and DP alleles that are commonly expressed in the general population.

**Figure 8:**
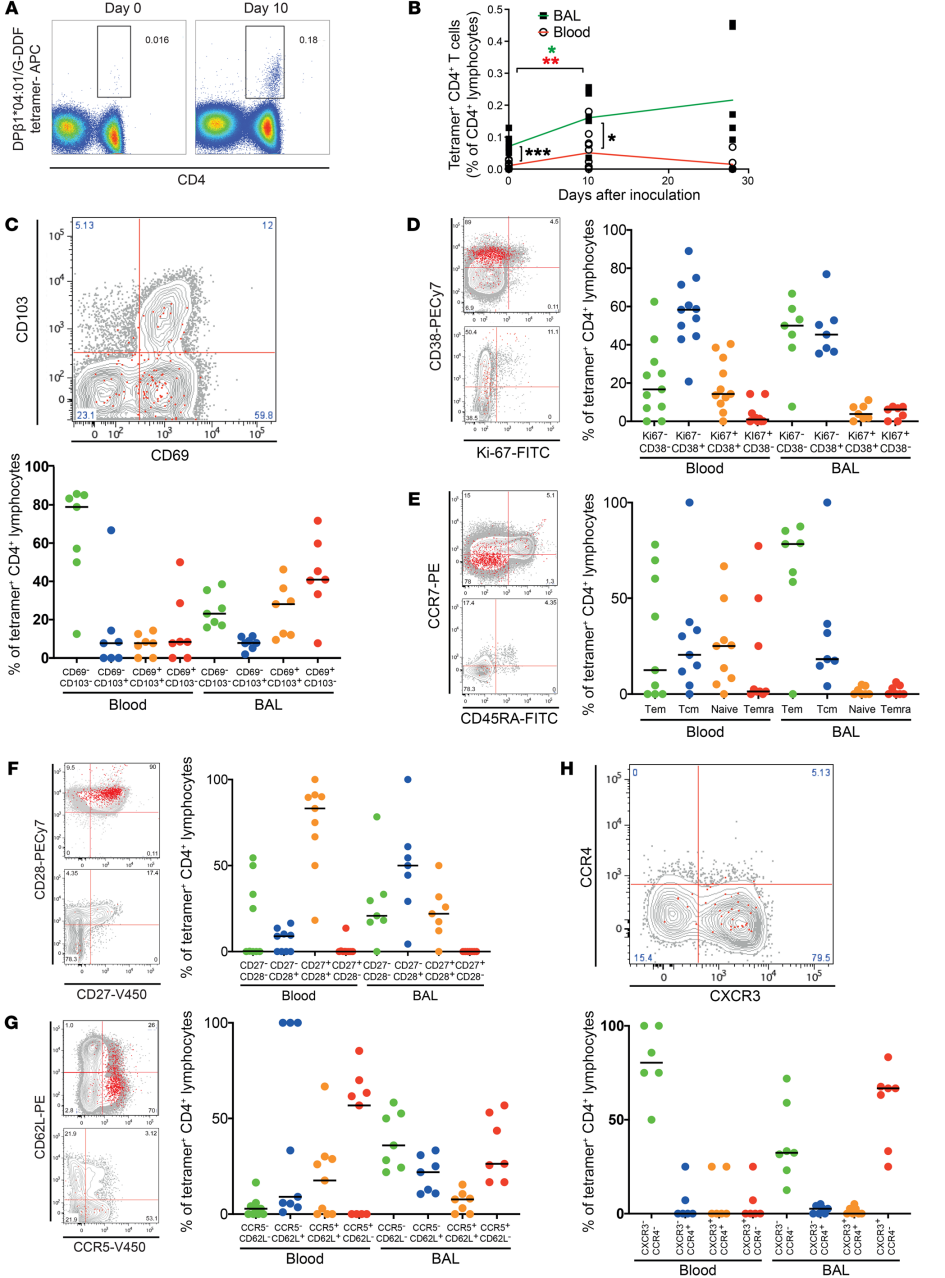
Epitope-specific CD4^+^ T cells preferentially accumulate and express CXCR3 in the airway during acute RSV infection. PBMCs from DPA1*01:03/DPB1*04:01–expressing individuals infected with RSV (*n* = 5) before infection and on day 10 were stained using DPB1*04:01/G-DDF tetramer and analyzed by flow cytometry. (**A**) FACS plots from 1 representative donor are shown, gated on CD3^+^ lymphocytes. (**B**) Cumulative data from blood (*n* = 11) and BAL (*n* = 7) are shown with *P* values for 2-tailed Mann-Whitney test comparing infected and uninfected groups, and 2-tailed Wilcoxon’s matched-pairs tests comparing time points. *P < 0.05, **P < 0.01, ***P < 0.001. PBMCs and BAL cells were costained with phenotypic markers of (**C**) T cell resident memory (CD69 and CD103), (**D**) proliferation/activation (Ki-67 and CD38), (**E**) memory subsets (CD45RA and CCR7), (**F**) costimulation (CD27 and CD28), homing receptors (**G**) CCR5 and CD62L and (**H**) CXCR3 and CCR4. FACS plots from 1 representative donor are shown, gated on CD3^+^CD4^+^ T lymphocytes. Red dots represent tetramer^+^ cells, and gray contours show total CD4^+^ T cells. The median frequencies of each subset are shown. Where not indicated with asterisks, differences were not statistically significant.

*MHC peptide tetramer–labeled RSV-specific CD4^+^ Trmcells predominantly express CXCR3*. To track RSV epitope-specific CD4^+^ T cells during infection, MHC-peptide tetramers were generated using F-EFY and G-DDF and the top HLAs to which they were restricted. Initially, thawed PBMCs from 6 subjects at baseline and 4 subjects on day 10 after infection were stained with class II–associated invariant peptide tetramers (CLIP negative control) and G-DDF/DPB1*04:01 tetramers using optimized conditions (Figure 8A and Supplemental Figure 8A for gating strategy). Nonspecific binding to CD4^+^ T cells by the CLIP tetramer was negligible throughout (Supplemental Figure 8B). Preinfection, G-DDF–specific CD4^+^ T cells were rare (mean, 0.019% ± SEM 0.003; Figure 8B). However, in line with the earlier total CD4^+^ T cell and ELISpot data, there was a significant increase (*P* = 0.0436) in G-DDF–specific CD4^+^ T cells in blood on day 10 following infection (mean, 0.108% ± SEM 0.026). Thus, epitope-specific CD4^+^ T cells could be detected in blood ex vivo following RSV infection using this tetramer without further enrichment.

These 5 newly created tetramers were then used to label RSV-specific CD4^+^ T cells from a separate cohort of 8 challenged volunteers, of whom 3 were infected (Figure 1B). When participants expressed more than 1 appropriate HLA allele, blood and BAL samples were stained with each corresponding tetramer. Although epitope-specific CD4^+^ T cells against RSV were rare even in the airway before infection (Figure 8B), the frequency of RSV-specific CD4^+^ T cells was significantly higher in BAL than blood at rest (*P* = 0.0001). Following infection, their frequency in BAL also increased significantly (*P* = 0.0312), remaining enriched compared with blood (*P* = 0.0156). On day 28 after infection this population had contracted, although in some individuals, BAL CD4^+^ T cells continued to rise in frequency during convalescence.

The low frequencies precluded accurate phenotypic analysis of tetramer-labeled cells before infection. However, the expanded populations on day 10 after infection enabled robust phenotypic analysis of epitope-specific CD4^+^ T cells in both blood and BAL. The majority of tetramer^+^CD4^+^ T cells in BAL, in contrast with blood, expressed the Trm cell marker CD69 alone (median, 40.9% of tetramer^+^ cells; IQR, 59.8–33.3) with a proportion (median, 28.1%; IQR, 36.4–12) also expressing CD103 (Figure 8C). Costaining for CD38 and Ki-67 showed the majority of RSV-specific CD4^+^ T cells in blood to express CD38 alone (median, 58.8%; IQR, 71.4–44.5), indicative of activation but not proliferation, with a smaller proportion coexpressing CD38 and Ki-67 (median, 14.3%; IQR, 33.3–9.28; Figure 8D). This was even more marked in BAL, where only 3.8% (median; IQR, 7.69–1.58) expressed both CD38 and Ki-67, suggesting that actively proliferating RSV-specific cells were limited in the airway. Because CD69 is also a marker of activation, we analyzed coexpression of this marker with CD38 and found coexpression in a minority of tetramer^+^CD4^+^ T cells in uninfected individuals on day 10. However, a significant majority expressing both markers in infected individuals was found at this acute time point (Supplemental Figure 8C), highlighting the difficulty in interpretation of CD69 alone as a marker of Trm cells in a dynamic system.

Analysis of memory subsets using CD45RA and CCR7 showed that the majority of RSV-specific blood CD4^+^ T cells at this time point displayed a memory phenotype (Figure 8E) in contrast to the overall CD4^+^ population, which was dominated by naive cells (Figure 4B). In BAL, this was even more skewed, with no CD45RA^+^ cells and 78.3% (median; IQR, 85.2–58.5) displaying an effector/effector memory phenotype. Strikingly, while the majority of these cells in blood coexpressed CD27 and CD28 (median, 83.2%; IQR, 90.6–58.4), in BAL, the majority had downregulated the expression of CD27 alone (median, 50%; IQR, 61–29.2) or both CD27 and CD28 (median, 20.8%; IQR, 33.3–17.1) indicative of more advanced T cell differentiation (Figure 8F). RSV-specific CD4^+^ T cells did not express the cytotoxicity marker perforin and minimally expressed granzyme B (Supplemental Figure 8D).

During infection, T cells migrate from lymphoid tissues to areas of inflammation along chemokine gradients. Therefore, almost all RSV-specific CD4^+^ T cells in blood had upregulated the chemokine receptor CCR5, with most also downregulating CD62L expression (median, 58.5; IQR, 68.7–0.0) and a similar pattern was seen in BAL (Figure 8G). Furthermore, the importance of CXCL10, which correlated strongly with activated CD4^+^ T cell recruitment in BAL (Figure 3D), was further supported by analysis of its receptor CXCR3, which was expressed by the majority of RSV-specific CD4^+^ T cells in BAL (median, 66.7% ± IQR, 67.6–33.3) but not blood (Figure 8H) and the absence of CCR4 expression, which in mice enables lung homing via its ligands CCL17 and CCL22 (37).

Thus, the expanded T cell population during acute infection in volunteers allowed us to define the breadth of immunodominant CD4^+^ T cell epitopes in RSV and their HLA restrictions to generate potentially novel MHC class II tetramers. Using this system, we also showed the distinct patterns and dynamics of RSV-specific lung-resident memory CD4^+^ T cells, which underwent greater proliferation and differentiation to a lung-homing phenotype in response to RSV infection compared with nonresident counterparts in peripheral blood.

## Discussion

In this study, we took advantage of the unique features of experimental human RSV infection to systematically analyze the T cell response and, in particular, to examine CD4^+^ T cells in the human lung. Previous analyses using the same system demonstrated a significant correlation between nasal RSV-specific IgA and protection from infection (32), whereas CD8^+^ T cells in the lung correlated with reduced disease severity (34). These data partially explain the heterogeneity of clinical outcome following human RSV challenge, but the lack of appropriate tools has limited our ability to measure RSV-specific CD4^+^ T cells, which are likely to also play a critical role in antiviral immunity. Therefore, functional mapping of antigen-specific CD4^+^ T cells was used to identify several previously unrecognized epitopes and immunodominant responses against the major surface glycoproteins F and G. Novel tetramers were then created to determine the dynamics and phenotype of RSV-specific CD4^+^ T cells in the human lung.

Analysis of CD4^+^ T cell activation and proliferation in blood showed a pattern similar to that seen in CD8^+^ T cells during RSV infection (34). Compared with blood, RSV-specific CD4^+^ T cells were more frequent in the airway, although the overall CD4^+^ T cell response to RSV infection was substantially smaller than CD8^+^ T cells. Distinct patterns of CD4^+^ T cell composition were also seen, with significant increases in Treg cells in BAL on infection that were not seen in blood. Murine Tregs have been shown to restrain RSV-induced immunopathology including via lung-specific mechanisms mediated by granzyme B (38). However, whilst pediatric RSV bronchiolitis is associated with lower frequencies of Tregs in blood, it remains unclear whether this is a reason for more severe disease or the result (39). Our data show that in the adult pulmonary response to RSV infection, Tregs are preferentially generated in the airway alongside conventional effector cells, presumably acting locally to prevent dysregulated inflammation.

Although most RSV-specific CD4^+^ T cells coexpressed IFN-γ and TNF, few exhibited the polyfunctionality characterized by more than 2 cytokines (40, 41). This finding paralleled the relatively modest polyfunctionality of RSV-specific CD8^+^ T cells compared with those induced by influenza (34). Furthermore, few RSV-specific CD4^+^ T cells expressed the proliferation marker Ki-67 compared with CD8^+^ counterparts. Although directly comparable studies are not available, Ki-67 expression by CD4^+^ T cells has been shown with influenza vaccination (42), suggesting that its absence might be particular to RSV infection. RSV is known to encode a number of immunomodulatory proteins that impair antigen presentation (43) and inhibit type I interferon (44, 45). It remains to be seen whether antigen delivery by vaccines without these immunomodulatory proteins can overcome these functional defects.

Due to the low resting frequencies of RSV-specific CD4^+^ T cells, previous studies have focused on investigation of selected proteins by immortalization of T cell clones or in vitro expansion of PBMCs (29, 30, 46, 47). The limitations of these techniques rest in the inevitable bias introduced. In contrast, the expanded populations of CD4^+^ T cells shortly after peak viral load following experimental infection showed that the majority of responses shared by multiple individuals were directed against the internal proteins NS1, NS2, and M (recognized by CD8^+^ T cells) and the surface glycoproteins F and G (mainly recognized by CD4^+^ T cells), all of which are encoded early in the genome and expressed in relative abundance. Substantial interindividual variation was apparent, however, with differences in size and breadth of response in different subjects, presumably related to divergent HLA haplotypes and RSV infection history. In influenza, previous challenge studies have also shown diverse T cell responses directed against both surface and internal proteins, with similar magnitude and dynamics (2). HLA-DR1 transgenic mice have also been used to model human influenza-specific CD4^+^ T cell responses (48) and broadly target both surface and internal proteins. Further analysis of influenza-specific responses in challenge models will help elucidate whether this is due to differences between mice and humans or influenza and RSV.

The detection of CD4^+^ T cell responses against F proteins in all study participants is reassuring because all current RSV vaccine candidates include F protein as the main antigen (31). However, with a combination of inference from each subject’s HLA type, in silico prediction and measurement of MHC-peptide binding affinity in vitro, the immunodominant F-EFY epitope was shown to be restricted to HLA-DR4, -DR9, and -DR11, which have a combined allelic frequency of only 15% in European populations (49). In contrast, the G-DDF epitope was restricted to HLA-DPA1*01:03/DPB1*02:01 and -DPA1*01:03/DPB1*04:01, the allele frequency of which is 55%. Recent studies have suggested that the addition of G protein in vaccine candidates may enhance protection by stimulating immune responses that modulate immunopathogenesis, and these findings further support its addition due to the presence therein of this widely recognized immunodominant epitope that we speculate might enhance CD4^+^ T cell help (50, 51).

The use of MHC class II tetramers presents considerable technical challenges (52), but we showed that sensitive tetramer reagents could be generated by identifying immunodominant epitopes and their HLA restriction in this system. In BAL, most RSV-specific CD4^+^ T cells expressed the lectin CD69, which in humans is the only well-recognized marker of CD4^+^ Trm cells (53). However, our data showed substantial coexpression of this marker with CD38, commensurate with its role as an early activation marker and highlighting the need for further, more specific, CD4^+^ Trm cell markers that are not confounded by cellular activation in a dynamic system. Interestingly, a proportion of CD69^+^CD4^+^ T cells also expressed the integrin CD103 (a canonical marker of CD8^+^ Trm cells), that has only been described previously in a subset of CD4^+^ Trm cells in skin (54). Furthermore, tetramer^+^CD4^+^ T cells in BAL were predominantly highly differentiated effector/effector memory Trm cells expressing CCR5 and CXCR3, features that had previously been indiscernible by analysis of the total CD4^+^ population. The expression of CXCR3 by RSV-specific CD4^+^ T cells in the airway but not in blood suggests that these are preferentially recruited to the lung. Unlike other tissues, where T cells may be directed by specific integrins and chemokine receptors, no specific combination of homing markers has been defined that imprints T cells destined for lung homing (55, 56). Although in influenza-infected mice, expression of CCL17 and CCL22 by airway epithelial cells is associated with imprinting of CCR4^+^CD4^+^ T cells by lung dendritic cells, our findings suggest that this does not occur with human RSV. Instead, our data show that CXCL10 concentrations in bronchial lining fluid correlated strongly with the frequency of activated CD4^+^ T cells in BAL, which predominantly express CXCR3. The CXCL10/CXCR3 axis has been previously implicated in protection against RSV-induced lung pathology in mice via dendritic cells and CD8^+^ T cells (57). In humans, little is known about CXCL10/CXCR3 in the context of respiratory infections. However, they are upregulated in patients with several chronic and/or systemic infections (58, 59), acute respiratory distress syndrome (60), and in the blood of children infected with respiratory viruses (61). Our data imply that the CXCL10/CXCR3 axis may be particularly important in trafficking of RSV-specific CD4^+^ T cells to the lung and support the development of this as a biomarker and adjuvant for mucosal vaccination.

Progress in understanding of human CD4^+^ T cells as well as enhancing efforts toward successful RSV vaccine development requires detailed understanding of their epitope specificity and mechanisms of generation, recruitment, and maintenance. By elucidating the breadth of the human cell-mediated response to RSV, we have identified the antigenic targets required to enlist T cell help; reagents with which to investigate RSV-specific CD4^+^ T cells at a single-cell level; and pathways through which adjuvants might enhance CD4^+^ Trm cell recruitment to the lung. We anticipate that these will accelerate our understanding of CD4^+^ T cells in enhancing protection, accelerating the generation of robust anti-RSV immunity through vaccination.

## Methods

### Study design.

Forty-nine healthy, nonsmoking adults 18 to 55 years of age were enrolled during summer months (i.e., outside the RSV season), inoculated with 1 × 10^4^ PFUs of RSV A M37 by i.n. drops and quarantined for 10 days. They returned on days +14 and +28 for sampling in the convalescence period. An additional 8 volunteers were enrolled subsequently for further phenotypic analysis of RSV-specific CD4^+^ T cells. At all time points, subjects were asked to complete self-reported symptom diary cards based on a modified Jackson scoring system (32). Systemic, upper respiratory, and lower respiratory tract symptoms were graded 0–3 in which 0 = absent; 1 = mild; 2 = moderate; and 3 = severe. Nasal lavage samples were collected daily and viral load assayed by quantitative PCR as previously described (32). Subjects were regarded as RSV infected if virus was detected by PCR of nasal lavage on at least 2 days between day +2 and day +10. Twenty-four individuals underwent bronchoscopy prior to inoculation, 7 to 10 days after inoculation, and during convalescence on day +28 as previously described (34). All subjects were genotyped for HLA class II (DR, DP, and DQ) using sequence-based typing (Anthony Nolan typing services).

### PBMC isolation.

PBMC isolation was performed by density centrifugation using Histopaque 1077 (Sigma-Aldrich) according to the manufacturer’s protocol. Isolated PBMCs were used immediately or cryopreserved in FCS (Gibco Life Technologies) with 10% DMSO in liquid nitrogen.

### Bronchial sampling.

BAL, bronchial brushings, bronchoabsorption, and endobronchial biopsies were obtained before infection, day 7 to 10, and day 28 time points from all participants who underwent bronchoscopy. BAL samples were obtained using up to 200 ml of saline via a fiberoptic bronchoscope, filtered through a 70-μm strainer, and resuspended in ACK buffer to lyse red cells. Cells were counted using trypan blue and used immediately. Bronchial brushings were obtained using disposable cytology brushes (Olympus EndoTherapy) with the bronchoscope positioned proximal to the right lower lobe. After gently brushing the bronchial wall, brushes were retracted and clipped off into warm RPMI/10% FCS. Following mechanical dissociation, bronchial brushing cells were centrifuged and resuspended in Trizol (Thermo Fischer Scientific) and stored at –80°C. Bronchial lining fluid was collected using disposable bronchoabsorption probes (Hunt Diagnostics) with the synthetic absorptive matrix strip laid on the segmental bronchial wall for 30 seconds before retraction. This was eluted in 100 μL of PBS containing 1% bovine serum albumin (w/v), 0.5% Triton-X (v/v), and 0.05% sodium azide (w/v) (all Sigma-Aldrich) and stored at –80°C for later analysis by Meso Scale Discovery (Meso Scale Diagnostics) according to the manufacturer’s instructions.

### Endobronchial biopsy sampling and immunohistochemistry.

Endobronchial biopsies were performed using disposable biopsy forceps with jaws closed at the interbronchial carina before retracting. Tissue was fixed immediately in 4% paraformaldehyde and paraffin embedded. CD4^+^ T cells were identified by staining with mouse anti-CD4 (Dako) at 1:100 dilution using the EnVision peroxidase staining method (Dako) as previously described (62). Slides were coded to avoid observer bias and assessed using a Leitz Dialux 20 light microscope and Image 1.5 software. Total epithelial and subepithelial areas of 2 to 3 bronchial biopsies were counted at each time point. Cell counts were expressed as the number of cut cell profiles with visible nucleus per mm^2^ of subepithelium and per 0.1 mm^2^ of epithelium. The coefficient of variation for repeat counts of positive cells by a single observer ranged from 5% to 6%.

### Flow cytometry.

BAL cells, whole blood, or PBMCs were stained with antibodies (Supplemental Table 5) for 15 minutes in the dark at 4°C, washed, and the cells were resuspended for flow cytometric analysis. For intracellular cytokines, surface-stained cells were incubated with a BD Fixation/Permeabilization kit for 30 minutes and washed with BD Permeabilization/Wash buffer. Monoclonal antibodies against cytokines of interest were added, and cells were incubated in the dark for approximately 45 minutes, washed, and analyzed on a BD LSRFortessa. The following reagents were obtained through the NIH Tetramer Core Facility: EFY/HLA-DR4, EFY/HLA-DR9, EFY/HLA-DR11, DDF/HLA-DPA1*01:03/DPB1*03:01, and DDF/DPA1*01:03/DPB1*04:01. Prior to tetramer staining, cells were treated with dasatinib (Axon Medchem) for 30 minutes at 37°C at a final concentration of 50 nM. Cells were washed and stained with class II tetramer of interest and its relevant CLIP tetramer negative control. Tetramer staining was carried out at 2 μg/mL for 15 minutes in the dark at 37°C. Following class II tetramer staining, cells were stained for surface and intracellular markers as described previously.

### Total RNA extraction and RT2 Profiler qPCR array.

To extract total RNA from bronchial brushings, 0.2-mL chloroform per 1 mL TRIzol was added, shaken vigorously, then centrifuged. The aqueous phase was transferred, and an equal volume of 70% ethanol was added. RNA was then extracted using RNeasy Mini kit (Qiagen) according to the manufacturer’s instructions. Total RNA was quantified by NanoDrop 2000 (Thermo Fisher Scientific), and 100 ng per sample was used for cDNA synthesis with the RT2 First Strand and RT2 PreAMP cDNA Synthesis kits (Qiagen). RT2 SYBR Green Mastermix (Qiagen) was added to the amplified products and loaded on RT2 Profiler PCR Human Cytokines and Chemokines arrays. These were run on a real-time cycler (Applied Biosystems 7500 Fast Real-Time PCR System) using the RT2 Profiler manufacturer’s cycling conditions.

### Generation of RSV peptide library and pools.

A peptide library of 15-mers with each overlapping its neighbor by 10 amino acids was generated using the RSV M37 sequence (34). Peptides synthesized at greater than 80% purity (Mimotopes) were pooled for use in epitope-mapping studies using a previously described matrix method (41) at a final concentration of 1.470 μg/mL. Peptides were stored in DMSO at –20°C.

### IFN-γ ELISpot assay.

IFN-γ ELISpot was performed using 2 × 10^5^ PBMCs in triplicate stimulated with peptide pools or single peptides (10 μg/mL). Plates were incubated for 18 hours at 37°C and developed for 15 minutes using the BCIP/NBT-plus substrate following manufacturer’s instructions (Mabtech). Spots were counted using AID ELISpot software (Autoimmun Diagnostika GmbH), with positive wells containing more than 20 spots per 1 × 10^6^ cells.

### In vitro peptide binding affinity.

Peptide binding affinity for HLA class II molecules was measured using in vitro quantitative classical competition assays, as previously described (63). Briefly, each peptide of interest was incubated in the presence of purified MHC, an allele-specific high-affinity radiolabeled standard peptide, and a cocktail of protease inhibitors. Following incubation, MHC-bound radioactivity was determined by capturing MHC/peptide complexes on MHC locus–specific mAb-coated Lumitrac 600 plates (Greiner Bio-one) and measuring bound cpm using the TopCount (Packard Instrument Co.) microscintillation counter. The concentration of peptide yielding 50% inhibition of binding of the radiolabeled peptide was then calculated (64, 65). Each competitor peptide was tested at 6 different concentrations covering a 100,000-fold range and in more than 3 independent experiments. The unlabeled version of the radiolabeled probe was used as a positive control.

### CD4^+^ T cell proliferation assay.

Thawed PBMCs were labeled with CFSE (final concentration 2.5 μM), and 1 × 10^6^ cells were cultured with peptide of interest (10 μg/mL) supplemented with human recombinant IL-7 (R&D Systems) at 25 ng/mL on days 0, 7, and 10 and human recombinant IL-2 (Thermo Fisher Scientific) at 50 IU/mL on days 3, 7, and 10. Cultures were incubated at 37°C for 7 days.

### Magnetic-activated cell sorting.

Magnetic-activated cell sorting kits were purchased from Miltenyi Biotech. CD8^+^ T cell depletion from PBMCs was performed according to the manufacturer’s instructions using CD8 MicroBeads. An aliquot of the eluted samples was stained with anti-CD3, -CD4, and -CD8 antibodies for a purity check using flow cytometry. The purity of CD8^+^ cell depletion was at least 95% throughout.

### Statistics.

Statistical analysis was performed using GraphPad Prism. Two-group comparisons were tested, depending on whether data were normally distributed, using Student’s *t* test or Mann-Whitney test (unpaired) and paired *t* test or Wilcoxon’s matched-pairs signed-rank test for paired data. Comparison between more than 2 groups were by 1-way ANOVA. For all tests, a value of *P* less than 0.05 was considered significant. *P ≤ 0.05, **P ≤ 0.01, ***P ≤ 0.001; NS, not significant.

### Study approval.

The study was approved by the UK National Ethics Service London–Fulham (study numbers 10/H0711/94 and 11/LO/1826). Written informed consent was obtained from all volunteers prior to inclusion in the study.

## Author contributions

CC, PJO, SLJ, A Sette, and BP designed and conceived the study. CC, MSH, AP, SP, ZG, and EB carried out the experimental infection study. CC, AP, ZG, EB, MP, JD, MA, EHCW, A Sykes, JDR, MBTT, PM, and OMK conducted the bronchoscopies. AG, CC, AJ, SA, SKU, SP, MSH, MK, SK, JZ, and JS performed the laboratory experiments. CC, AG, AJ, and PJO wrote the manuscript. All authors reviewed the manuscript prior to submission. Co–senior author position was determined by mutual agreement.

## Supplementary Material

Supplemental data

## Figures and Tables

**Table 2:**
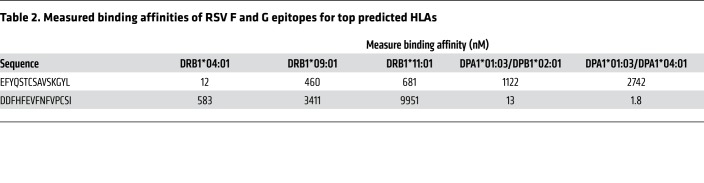
Measured binding affinities of RSV F and G epitopes for top predicted HLAs

**Table 1:**
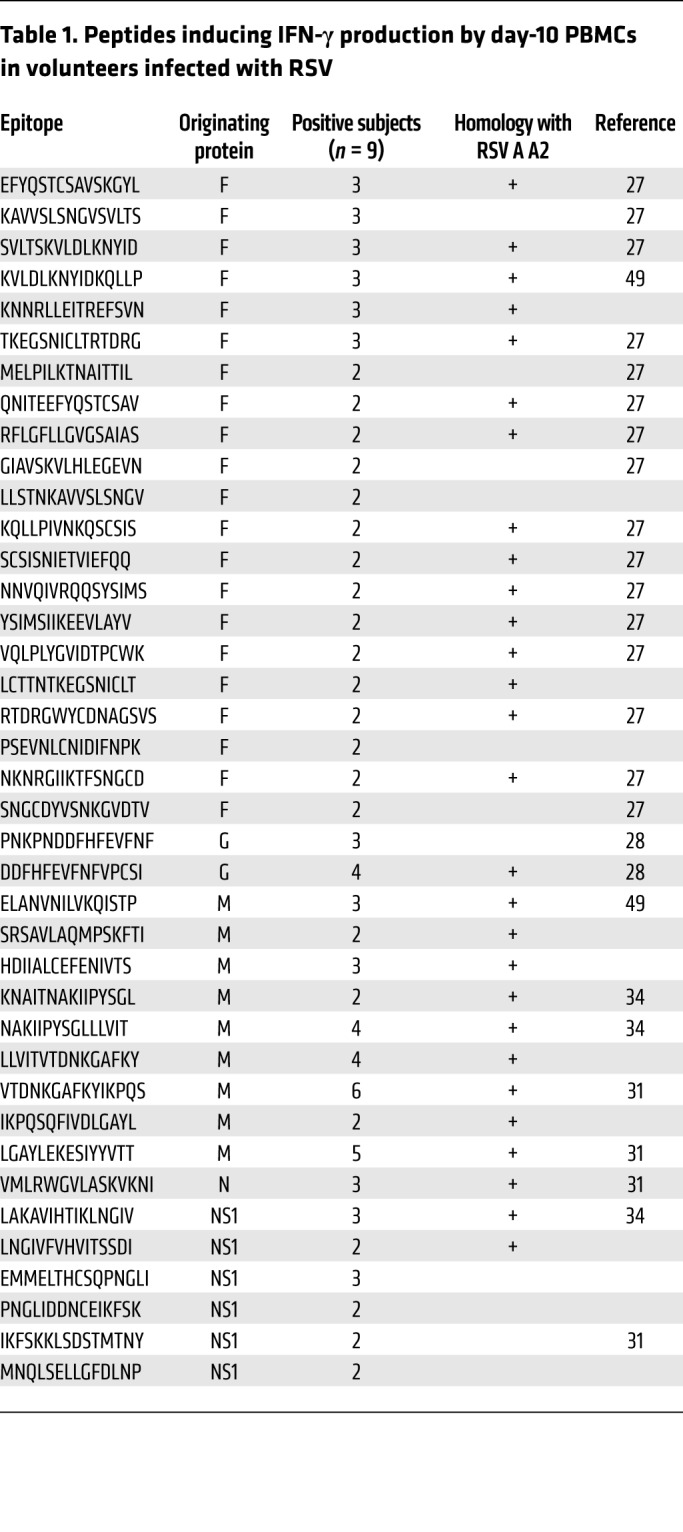
Peptides inducing IFN-γ production by day-10 PBMCs in volunteers infected with RSV

## Version 1 12/09/2019

Electronic publication

## Version 2 01/02/2020

Print issue publication
