# Supplementary material for: Epitope-specific airway-resident CD4+ T cell dynamics during experimental human RSV infection
Source: J Clin Invest. 2019 Dec 9;130(1):523–38. doi: 10.1172/JCI131696 (PMC6934186; doi:10.1172/JCI131696)
Supplement: Supplemental data [file jci-130-131696-s292.pdf]

Supplementary Figure 1

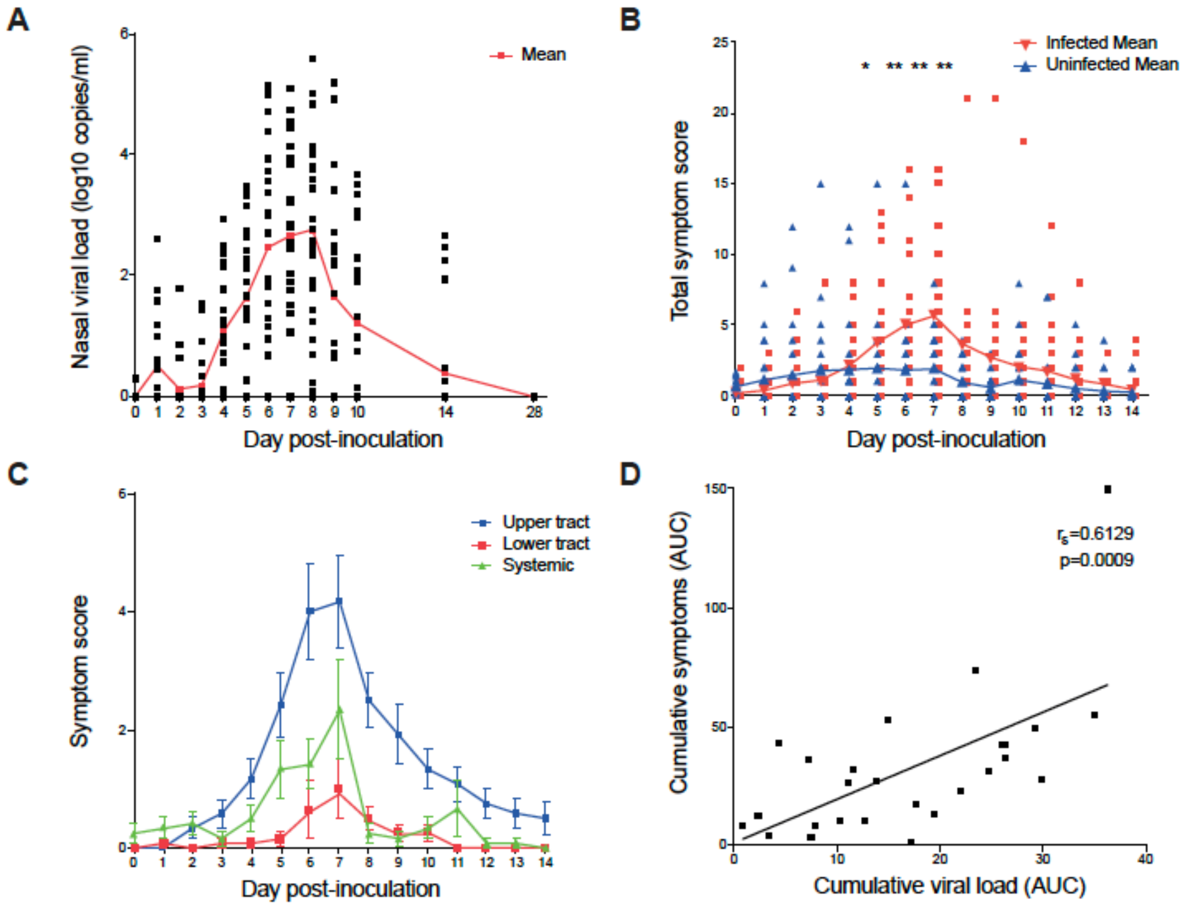

Supplementary Figure 1: Human RSV challenge induces a mild-moderate respiratory tract infection. Healthy adult volunteers were inoculated with RSV M37. (A) Viral load was measured by qPCR in nasal lavage. (B) Total or (C) upper tract, lower tract and systemic symptoms were calculated from self-complete symptom diaries. (D) Spearman's correlation between cumulative viral load and cumulative symptoms by trapezoidal area under the curse.

Supplementary Figure 2

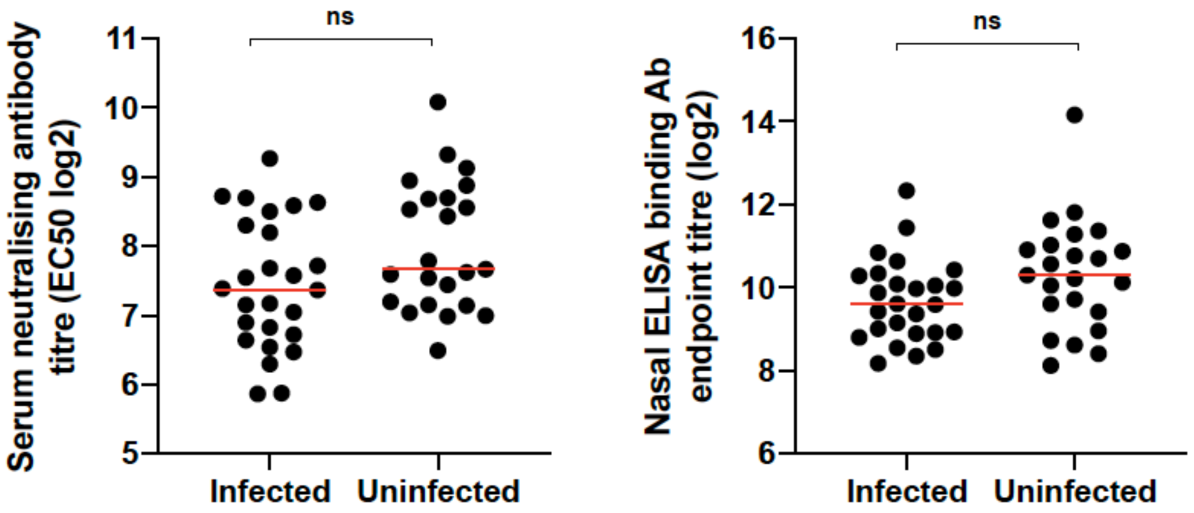

Supplementary Figure 2: Pre-inoculation antibody titres do not significantly correlate with protection. Serum and nasal lavage were collected from healthy volunteers prior to RSV challenge. (A) Serum neutralising antibodies were measured by classical plaque reduction neutralisation assay. (B) RSV-specific nasal IgA was measured by ELISA. Median and individual data points are shown. Wilcoxon signed-rank test was used to test for significant differences (ns = non-significant).

Supplementary Figure 3

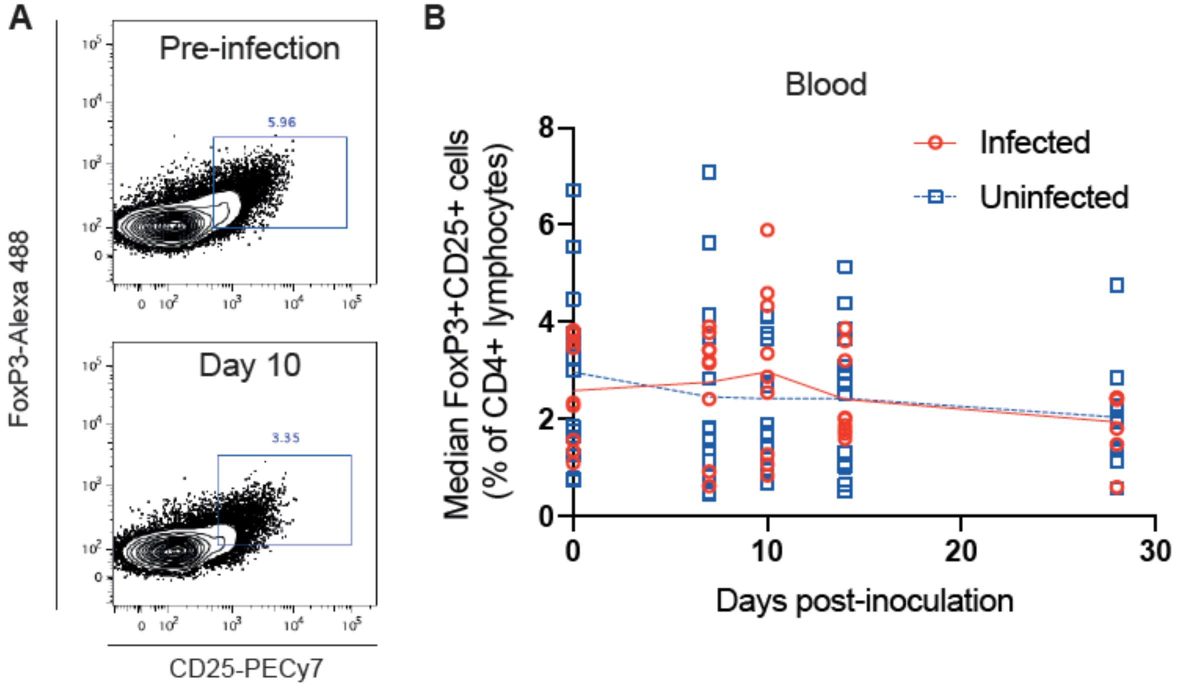

Supplementary Figure 3: Regulatory T-cell frequencies do not change in peripheral blood during experimental human RSV infection. CD4+FoxP3+CD25+ Treg cells were quantified by flow cytometry in whole blood. FACS plots from one representative donor are shown. Median and individual data points are shown in infected (red circles) and uninfected (blue squares) groups.

Supplementary Figure 4

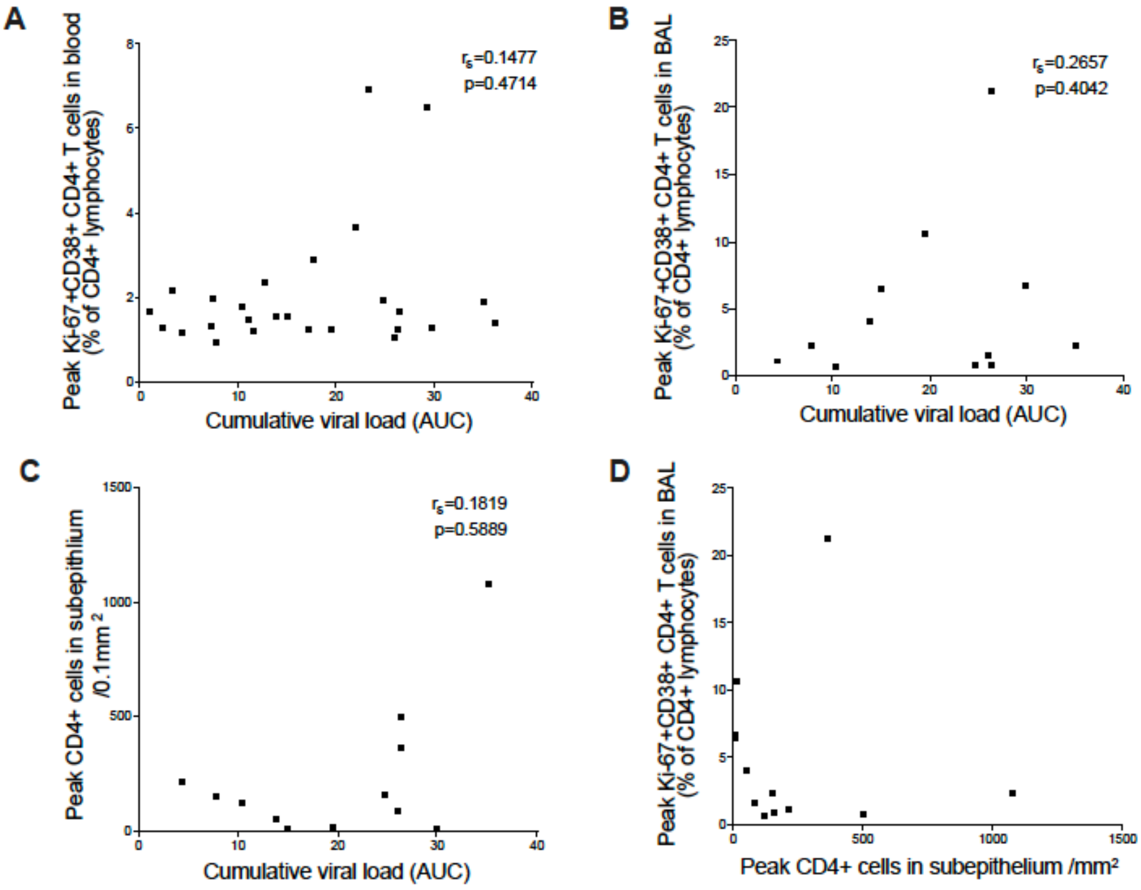

Supplementary Figure 4: Viral load does not correlate with activated CD4+ T-cell recruitment to the airway. Nasal lavage viral load was measured by qPCR and CD4+ T-cells quantified by flow cytometry and immunohistochemistry. Spearman's correlation between cumulative viral load and peak activated CD4+ T-cells in (A) blood and (B) BAL are shown. (C) Spearman's correlation between cumulative viral load and peak CD4+ cell numbers in the subepithelium of endobronchial biopsies is shown. (D) Spearman's correlation between peak CD4+ cell numbers in the subepithelium and activated CD4+ T-cells in BAL is shown.

Supplemental Figure 5

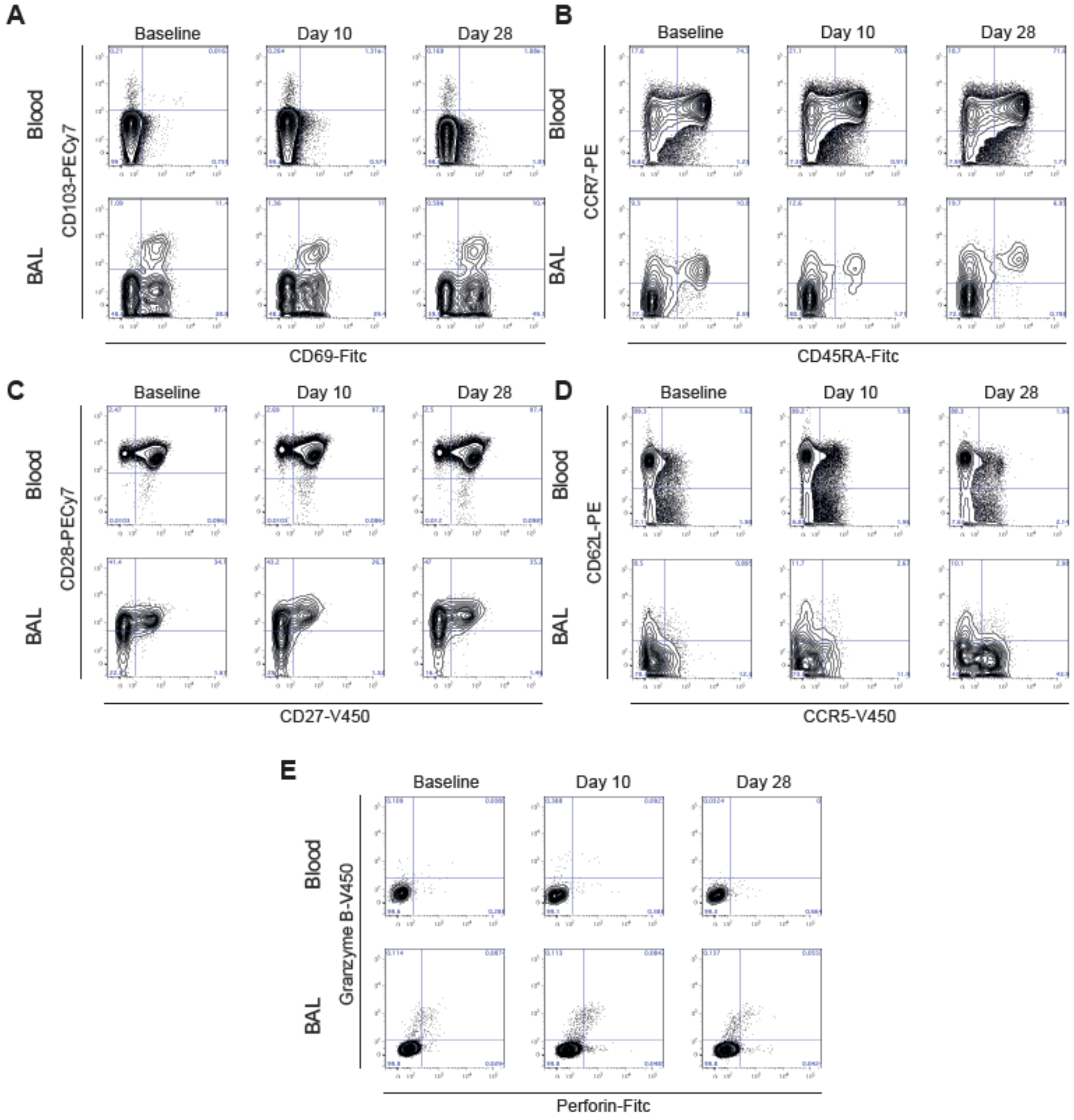

Supplementary Figure 5: CD4+ T-cells in blood and BAL are phenotypically distinct. Blood and BAL were obtained from healthy volunteers pre-infection, 10 and 28 days post-infection. CD4+ T-cells were phenotyped by flow cytometry using (A) anti-CD69 and CD103; (B) anti-CD45RA and CCR7; (C) anti-CD27 and CD28; (E) anti-CCR5 and CD62L; and (E) anti-perforin and granzyme B. Representative FACS plots from one individual are shown. Plots are gated on CD3+CD4+ lymphocytes.

Supplementary Figure 6

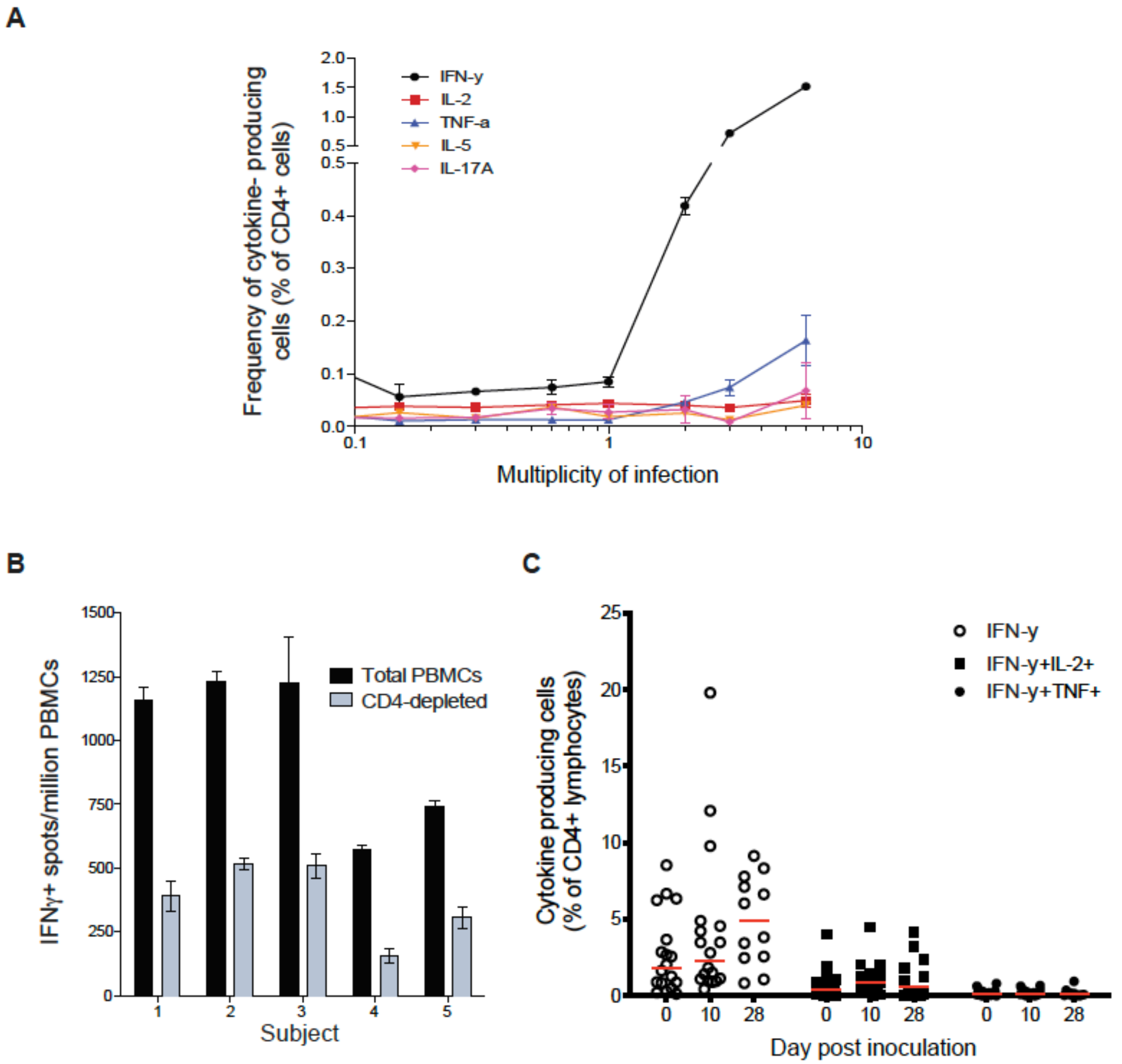

Supplementary Figure 6: CD4+ T-cells contribute <50% to the IFN-γ producing response to whole RSV. (A) Healthy donor PBMCs were cultured with live RSV up to an MOI of 6 and analysed by intracellular cytokine staining and flow cytometry. (B) Cytokine-producing CD4+ T-cells in response to PMA/ionomycin stimulation were enumerated using intracellular cytokine staining and flow cytometry. Median values are shown in red. (C) PMBCs at day 10 post-infection

and matched samples depleted of CD8+ cells by magnetic activated cell sorting from five subjects infected with RSV were stimulated with whole RSV for IFN-γ ELISpot.

Supplementary Figure 7

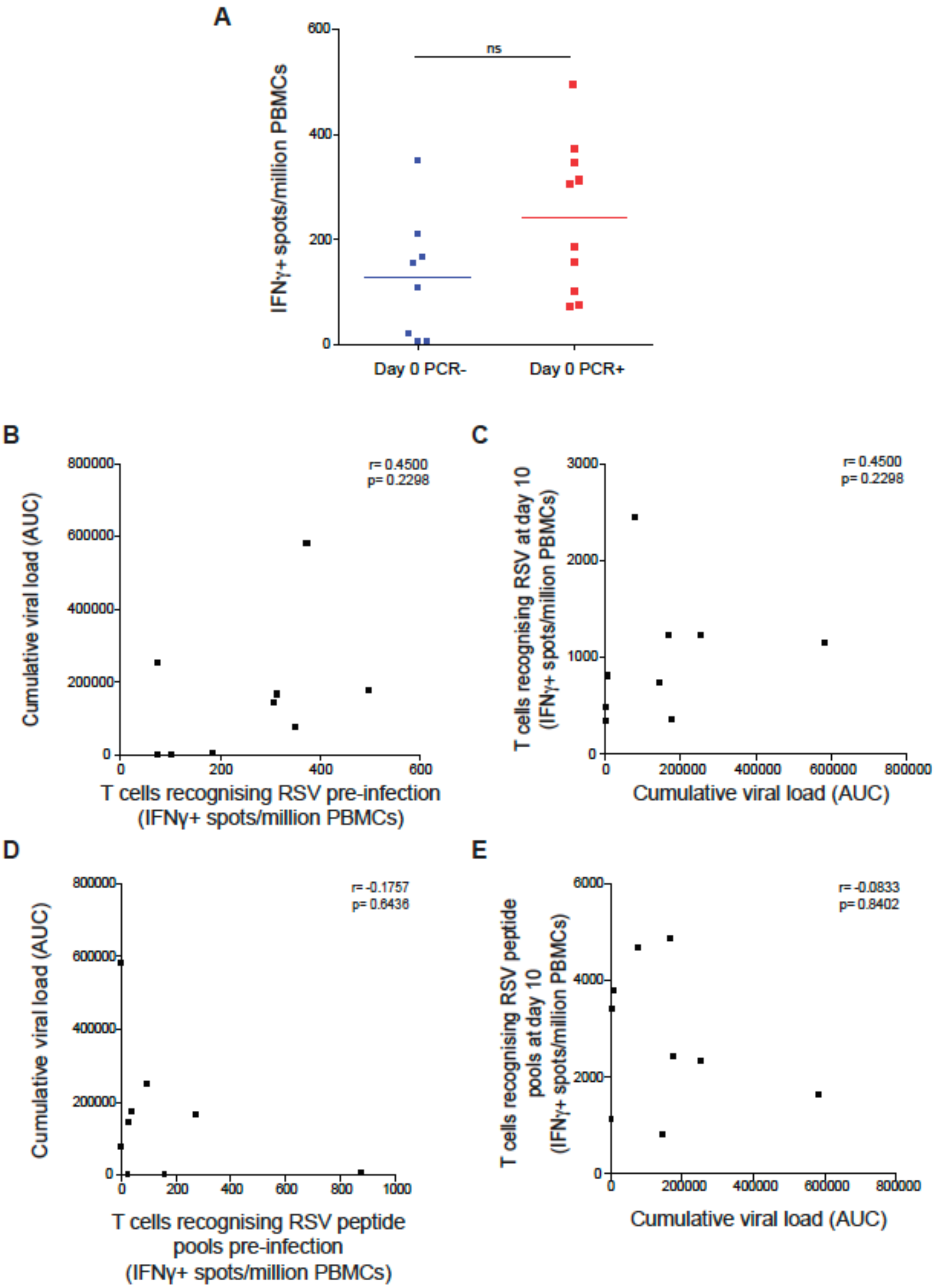

44

Supplementary Figure 7: RSV-specific memory T-cells in blood do not correlate with risk of infection or disease severity. (A) PBMCs from infected and uninfected volunteers challenged with RSV were assayed using whole RSV and IFN-γ ELISpot prior to inoculation. Mann–Whitney test was used to compare PCR- and PCR+ groups (ns = P>0.05). (B) Spearman's correlation between pre-existing IFN-γ producing cells recognizing whole RSV and cumulative viral load in RSV infected individuals. (C) Spearman's correlation between cumulative viral load and peak whole RSV-recognising T-cells. (D) Spearman's correlation between pre-infection RSV-specific T-cells producing IFN-γ in response to pooled peptides and cumulative viral load. (E) Spearman's correlation between cumulative viral load and RSV-specific T-cells at day 10 post-infection recognizing peptide pools.

Supplementary Figure 8

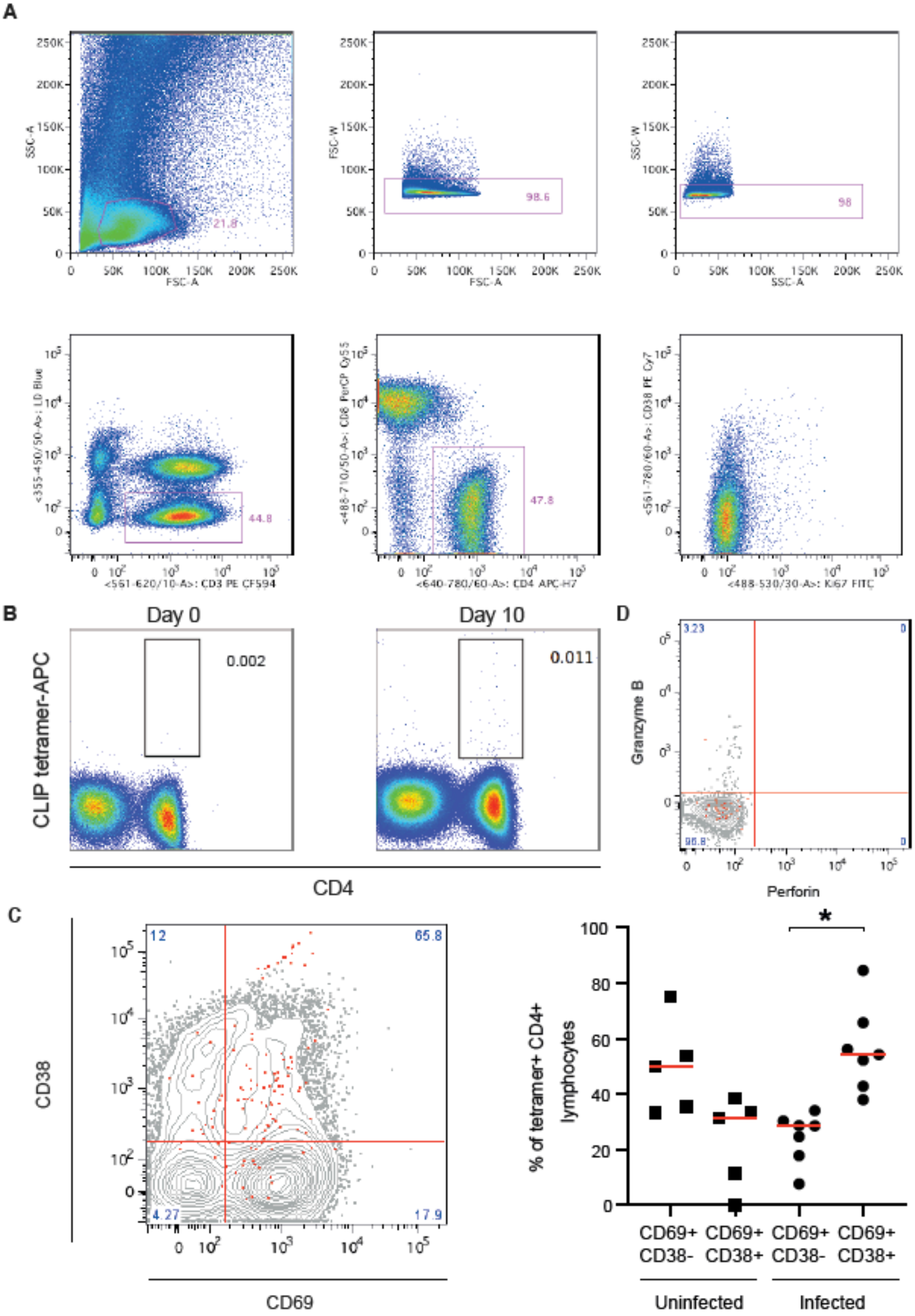

Supplementary Figure 8: CD69 and CD38 expression overlap in pulmonary CD4+ T-cells during RSV infection. (A) Gating strategy for whole blood and BAL cells co-stained with LIVE/DEAD Fixable Blue, anti-CD3, CD8, and phenotypic markers Ki-67 and CD37 for flow cytometric analysis is shown. CD4+ T-cells were gated on lymphocytes by size and granularity on FSC/SSC; singlets; live CD3+ cells; and CD4+CD8- cells. (B) Representative plot from a single individual of PBMCs stained with the negative control CLIP-tetramer at pre-infection and day 7 time-points. (C) BAL cells at day 10 post-RSV inoculation were stained with anti-CD3, CD4, tetramer, CD69 and CD38. FACS plot from one representative donor is shown. Red dots represent tetramer+ cells; grey contours show total CD4+ T-cells gated on CD3+CD4+ lymphocytes. Median and individual data points are shown. Wilcoxon signed-rank test was used as test of significance (*$p<0.05$). (D) Representative plot from a single individual of CD4+ T-cells in BAL stained with anti-perforin and granzyme B. Red dots represent tetramer+ cells and grey contours delineate total CD4+ T-cells.

	Uninfected (PCR-)	Infected (PCR+)
Number of subjects (%)	23 (47%)	26 (53%)
Median age (range)	20.5 (18-50)	23 (18-39)
Gender (M:F)	15:8	15:11
Ethnicity (%)		
White	17 (74%)	22 (85%)
Black	4 (17%)	1 (4%)
Chinese	0 (0%)	2 (8%)
Mixed	2 (9%)	1 (4%)

Supplementary Table 1: Demographic characteristics of 49 adult volunteers challenged with RSV M37

Gene Symbol	Ct value		Normalised Expression		p-value	log_2 (fold-change)
	Pre-infection	Day 7	Pre-infection	Day 7		
IL9	21.63	17.01	0	0.000008	0.285754	24.59
CXCL11	11.06	7.49	0.000468	0.005554	0.19623	11.86
CXCL10	6.44	3.11	0.011492	0.116177	0.227067	10.11
CXCL9	6.19	3.19	0.01373	0.109309	0.208887	7.96
CCL8	20.51	17.93	0.000001	0.000004	0.310658	5.96
CCL7	11.54	9.02	0.000335	0.00192	0.297041	5.72
CSF3	5.94	3.52	0.016325	0.087391	0.052278	5.35
CCL2	10.08	7.73	0.000921	0.00471	0.335833	5.11
IL3	16.15	14.18	0.000014	0.000054	0.261676	3.92
BMP2	12.3	10.46	0.000198	0.000712	0.080379	3.59
IL6	3.46	1.78	0.091089	0.290791	0.124984	3.19
LIF	5.37	3.96	0.024207	0.06419	0.219489	2.65
IL22	11.26	10.09	0.000408	0.000915	0.32275	2.24
CCL19	11.12	10.11	0.000449	0.000904	0.339014	2.01
IFNA2	20.13	19.24	0.000001	0.000002	0.346073	1.85
IL21	12.03	11.17	0.000239	0.000434	0.366206	1.82
TNF	3.37	2.54	0.097003	0.171875	0.37376	1.77
CX3CL1	4.94	4.14	0.03267	0.056783	0.077709	1.74
IL27	11.89	11.13	0.000264	0.000446	0.381172	1.69
CXCL2	5.33	4.68	0.024776	0.038994	0.71729	1.57
IL1B	0.84	0.19	0.559322	0.879089	0.318683	1.57
CXCL8	-3.01	-3.64	8.059093	12.461637	0.602527	1.55
CXCL13	7.97	7.37	0.003997	0.00604	0.429599	1.51
IL10	7.63	7.1	0.005054	0.007304	0.37459	1.45
CCL3	0.95	0.42	0.51722	0.745087	0.409618	1.44
CCL13	12.9	12.4	0.00013	0.000185	0.395268	1.42
CXCL1	0.46	0.05	0.725448	0.963338	0.802549	1.33
TNFSF10	1.17	0.76	0.445942	0.592017	0.131228	1.33
IL7	6.22	5.83	0.013429	0.01761	0.995958	1.31
CXCL5	6.5	6.13	0.011053	0.014316	0.78776	1.3
IL1A	5.34	5	0.024651	0.031305	0.772552	1.27
IL11	13.61	13.28	0.00008	0.000101	0.475677	1.26
IL23A	7.38	7.05	0.005994	0.00753	0.476232	1.26
BMP7	7.53	7.21	0.005417	0.006761	0.277519	1.25
IFNG	5.64	5.33	0.019991	0.02484	0.395899	1.24
IL15	5.63	5.32	0.020232	0.025022	0.331646	1.24
IL1RN	1.86	1.56	0.276282	0.338589	0.41276	1.23
CSF2	8.84	8.55	0.002181	0.002662	0.377761	1.22
VEGFA	2.05	1.77	0.241206	0.293947	0.859965	1.22

Gene Symbol	Ct value		Normalised Expression		p-value	log_2 (fold-change)
	Pre-infection	Day 7	Pre-infection	Day 7		
CCL24	8.9	8.62	0.002094	0.002533	0.695943	1.21
GAPDH	1.17	0.89	0.443476	0.538525	0.11166	1.21
CCL20	1.57	1.38	0.337211	0.383693	0.94099	1.14
IL12B	14.06	13.86	0.000059	0.000067	0.470365	1.14
LTA	13.35	13.15	0.000096	0.00011	0.457796	1.14
ACTB	-0.09	-0.24	1.063186	1.178613	0.601827	1.11
XCL1	11.72	11.65	0.000296	0.00031	0.662184	1.05
IL17F	11.93	11.9	0.000256	0.000261	0.991284	1.02
IL2	9.88	9.85	0.00106	0.001082	0.533326	1.02
SPP1	4.46	4.44	0.045289	0.045979	0.625137	1.02
B2M	-3.81	-3.82	14.022505	14.10931	0.847438	1.01
TNFSF13B	6.21	6.2	0.013547	0.013577	0.807881	1
CCL18	2.31	2.35	0.202285	0.19552	0.484352	-1.03
IL18	1.73	1.77	0.301166	0.29343	0.807613	-1.03
CXCL16	6.84	6.9	0.008755	0.008395	0.879405	-1.04
LTB	6.28	6.36	0.012868	0.012145	0.710007	-1.06
MIF	0.55	0.65	0.682494	0.635609	0.943363	-1.07
TGFB2	5.73	5.85	0.018777	0.017368	0.561834	-1.08
RPLP0	-1.28	-1.17	2.432534	2.246114	0.973619	-1.08
GPI	2.03	2.21	0.245632	0.215489	0.241764	-1.14
CSF1	3.6	3.81	0.08264	0.071456	0.686855	-1.16
CXCL12	9.8	10.06	0.001119	0.000936	0.533892	-1.2
CD40LG	11.15	11.43	0.00044	0.000363	0.676267	-1.21
HPRT1	4.01	4.33	0.062178	0.049715	0.174276	-1.25
CNTF	3.9	4.27	0.067051	0.051673	0.552649	-1.3
IL5	10.32	10.72	0.000781	0.000593	0.529541	-1.32
NODAL	9.18	9.58	0.001724	0.001302	0.859798	-1.32
IL24	9.06	9.51	0.001876	0.001373	0.221954	-1.37
CCL5	3.1	3.56	0.116573	0.084647	0.545451	-1.38
THPO	10.84	11.35	0.000546	0.000384	0.469365	-1.42
IL12A	5.56	6.08	0.021177	0.014795	0.221752	-1.43
IL13	11.03	11.55	0.000477	0.000333	0.8692	-1.43
FASLG	6.7	7.24	0.009639	0.006623	0.78236	-1.46
OSM	3.03	3.6	0.122088	0.08247	0.698886	-1.48
CCL1	14.31	14.88	0.000049	0.000033	0.928247	-1.49
PPBP	11.06	11.67	0.000469	0.000306	0.313889	-1.53
IL16	5.32	6.02	0.025023	0.015383	0.055609	-1.63
TNFRSF11B	9.77	10.5	0.001148	0.000688	0.31435	-1.67
MSTN	11.13	11.93	0.000446	0.000256	0.223378	-1.74

Gene Symbol	Ct value		Normalised Expression		p-value	log_2 (fold-change)
	**Pre-infection**	**Day 7**	**Pre-infection**	**Day 7**		
CCL17	7.28	8.13	0.00643	0.003576	0.241412	-1.8
BMP4	7.62	8.54	0.005088	0.002696	0.174492	-1.89
TNFSF11	9.58	10.52	0.001305	0.00068	0.097654	-1.92
BMP6	8.64	9.63	0.002508	0.001262	0.044137	-1.99
C5	5.33	6.34	0.02479	0.012304	0.025353	-2.01
ADIPOQ	18.45	19.57	0.000003	0.000001	0.152253	-2.19
IL4	8.89	10.15	0.002102	0.000881	0.569885	-2.39
IL17A	18.01	20.22	0.000004	0.000001	0.38372	-4.62
CCL11	13.23	15.79	0.000104	0.000018	0.185296	-5.9
CCL21	12.24	15.22	0.000207	0.000026	0.071645	-7.91
CCL22	7.59	11.4	0.005198	0.00037	0.136783	-14.04

Supplementary Table 2: Differential expression of soluble mediator genes between day 7 and day 0 by qPCR array

Sequence	HLA allele	Percentile rank
EFYQSTCSAVSKGYL	DRB1*04:01	0.42
	DRB1*09:01	1.72
	DRB1*04:05	2.62
	DRB1*04:04	3.13
	DRB1*11:01	4.2
	DRB1*08:02	8.08
	DRB1*07:01	12.75
	DRB1*01:01	13.04
	DRB1*03:01	18.64
DDFHFEVFNFVPCSI	DPA1*01:03/DPB1*02:01	0.21
	DPA1*01/DPB1*04:01	0.55
	DPA1*02:01/DPB1*01:01	2.23
	DQA1*01:01/DQB1*05:01	3.32
	DPA1*02:01/DPB1*05:01	5.14

Supplementary Table 3: Highest predicted binding affinities of immunodominant CD4+ T-cell epitopes from RSV

Subject ID	HLA-DRB1		HLA-DPA1		HLA-DPB1		HLA-DQA1		HLA-DQB1	
2	*11:04	*07:01	*01:03	*02:01	*04:01	*11:01	*02:01	*05:05	*02:02	*03:01
3	*15:01	*04:04	*01:03		*02:01	*04:02	*01:02	*03:01	*06:02	*03:02
9	*12:01	*07:01	*01:03		*04:01		*02:01	*05:05	*03:03	*03:01
1	*04:01	*10:01	*01:03		*02:01	*04:01	*01:01	*03:01	*05:01	*03:01
4	*07:01	*09:01	*01:03	*02:01	*03:02	*03:03	*02:01	*03:01	*03:02	*03:03
8	*11:01	*07:01	*01:03	*02:01	*03:01	*17:01	*02:01	*05:05	*03:03	*03:01

Supplementary Table 4: HLA types of 6 infected volunteers with CD4+ T-cell responses to G-DDF or F-EFY

Antibody	Company	Clone	Catalogue number	Dilution
CD3 PE CF594	BD	UCHT1	562280	1:300
CD4 APC H7	BD	SK3	641398	1:150
CD38 PE Cy7	BD	HB7	335825	1:150
Ki67 FITC	BD	B56	556026	1:150
Perforin FITC	BD	δG9	556577	1:30
Granzyme B V450	BD	GB11	561151	1:60
CD45RA FITC	BD	HI100	555488	1:60
CCR7 PE	BD	150503	560765	1:30
CD27 V450	BD	M-T271	560448	1:150
CD28 PE Cy7	BD	CD28.2	560684	1 :150
CCR5 V450	BD	2D7/CCR5	562121	1:60
CXCR3 PE CF594	BD	1C6	562451	1:30
CCR4 BV605	BD	1G1	562906	1:30
CD62L PE	BD	DREG-56	555544	1:60
CD69 FITC	eBioscience	FN50	11-0699-42	1:60
CD103 PE Cy7	eBioscience	B-Ly7	25-1038-42	1:150
CD8 PE Cy5.5	eBioscience	RPA-T8	45-0088-42	1:300
IFN-γ APC	BD	B27	554702	1:300
TNF PE Cy7	BD	MAb11	557647	1:60
IL2 FITC	BD	5344.111	340448	1:30
Anti-Human CD28/CD49d Purified	BD	L293, L25	347690	1:50

Supplementary Table 5: Antibodies used for flow cytometric staining
